# Deletion of neuronal *Idol* ameliorates Alzheimer's disease–related pathologies via APOE receptors

**DOI:** 10.1002/alz.70949

**Published:** 2025-12-12

**Authors:** Hande Karahan, Kelly Hartigan, Md Mamun Al‐Amin, Sutha K. John, Brianne McCord, H. R. Sagara Wijeratne, Dominic J. Acri, Daniel C. Smith, Luke C. Dabin, Hannah M. Rondon Cordero, Byungwook Kim, Do‐Hun Lee, Jungsu Kim

**Affiliations:** ^1^ Stark Neurosciences Research Institute Indiana University School of Medicine Indianapolis Indiana USA; ^2^ Department of Medical and Molecular Genetics Indiana University School of Medicine Indianapolis Indiana USA; ^3^ Medical Neuroscience Graduate Program Indiana University School of Medicine Indianapolis Indiana USA; ^4^ Department of Biochemistry and Molecular Biology Indiana University School of Medicine Indianapolis Indiana USA

**Keywords:** Amyloid beta, APOE, APOER2 (LRP8), IDOL (MYLIP), LDLR, VLDLR

## Abstract

**INTRODUCTION:**

Overexpression of the low‐density lipoprotein receptor (LDLR) is known to decrease apolipoprotein E (APOE) levels and alleviate amyloid beta (Aβ) pathology. We hypothesized that inhibiting the Inducible Degrader of LDLR (IDOL), an enzyme that ubiquitinates LDLR for degradation, would increase endogenous LDLR levels and attenuate amyloid pathology.

**METHODS:**

To investigate the cell‐type–specific role of IDOL, we generated *Idol* conditional knockout mice on an Aβ‐amyloidosis mouse model and performed biochemical, histological, and multi‐omics analyses.

**RESULTS:**

We demonstrated that neuronal, but not microglial, *Idol* deletion reduced amyloid accumulation and altered brain LDLR and APOE levels, indicating the critical role of neuronal IDOL‐LDLR in amyloid pathology. In addition, neuronal *Idol* deletion increased the levels of Reelin receptors important for synaptic function, and single‐nuclei RNA sequencing revealed significant changes associated with synaptic organization.

**DISCUSSION:**

Neuronal IDOL, but not microglial IDOL, plays a key role in Alzheimer's disease pathogenesis by regulating the levels of brain APOE receptors.

**Highlights:**

- Neuronal, but not microglial, *Idol* deletion reduces amyloid burden and modulates brain APOE and LDLR levels.
- Deletion of neuronal *Idol* increases the levels of APOER2 and VLDLR, the Reelin receptors, in the brain.
- Single‐nuclei RNA sequencing highlights the neuronal IDOL's impact on inhibitory neurons and synaptic organization.
- Targeting neuronal IDOL may provide multiple therapeutic benefits in Alzheimer's disease by modulating APOE receptors.

## 1 BACKGROUND

Advances in human genetics studies have led to the discovery of numerous genetic risk factors associated with late‐onset Alzheimer's disease (AD). However, apolipoprotein E (*APOE*) genotype remains the strongest known risk factor.^1^ This has led to a growing interest in understanding the role of APOE in the brain and its impact on AD pathogenesis. These efforts have resulted in identifying multiple mechanisms by which APOE influences AD‐related pathologies, including amyloid beta (Aβ) accumulation, tauopathy, neuroinflammation, and synaptic impairment.^2^ While the *APOE* ε4 isoform was identified as the greatest genetic risk factor for AD, the abundance of the APOE protein was also demonstrated to affect AD pathology. Both genetic deletion and pharmacological reduction of APOE reduced Aβ deposition in mouse models of AD.^3^, ^4^, ^5^, ^6^, ^7^, ^8^, ^9^ Of note, loss‐of‐function mutations in *APOE* delayed the onset of AD, regardless of the *APOE* genotype.^10^, ^11^ Therefore, we hypothesize that reducing APOE levels and identifying its endogenous regulators could generate new intervention opportunities for AD.

The APOE protein primarily mediates lipid transport in the brain.^12^ Although mainly secreted by astrocytes, *APOE* is also expressed in microglia, neurons, and oligodendrocytes, particularly under injury and degenerative conditions.^2^, ^13^ APOE binds to cell surface receptors, such as low‐density lipoprotein receptor (LDLR), LDLR‐related protein 1 (LRP1), very low‐density lipoprotein receptor (VLDLR), and apolipoprotein E receptor 2 (APOER2 or LRP8).^2^, ^14^, ^15^ Modulating these receptors affects Aβ pathology. For example, *Ldlr* overexpression markedly reduces amyloid plaque deposition.^16^ Because APOE co‐localizes with Aβ,^17^ the reduced amyloid deposition in *Ldlr*‐overexpressing mice may stem from the interaction between APOE and Aβ and their subsequent uptake via these receptors. Indeed, overexpression of *Ldlr* decreased both APOE and Aβ levels by enhancing Aβ clearance in amyloidosis mouse models.^16^, ^18^ These findings suggest that increasing brain LDLR levels could reduce APOE and Aβ, offering a potential therapeutic strategy for AD.

To increase endogenous LDLR levels, we targeted the inducible degrader of LDLR (IDOL, also known as MYLIP). IDOL is an E3 ubiquitin ligase that ubiquitinates LDLR, thereby triggering its lysosomal degradation.^19^ Our prior work using a conventional global *Idol* knockout (KO) model demonstrated that *Idol* deletion was sufficient to increase endogenous LDLR levels, coinciding with reduced amyloid plaque burden and neuroinflammation in an Aβ‐amyloidosis mouse model.^20^ However, because this approach targeted all cell types in every tissue, the cell type–specific mechanisms underlying amyloid reduction remained unknown. In this study, we aimed to identify the cellular mechanisms underlying the beneficial effects of *Idol* deletion in the context of Aβ deposition. Because *Idol* is expressed predominantly in microglia and neurons in the brain,^20^ we generated microglia‐ and neuron‐specific *Idol* KO mouse models and crossbred these mice with the 5XFAD Aβ‐amyloidosis model to investigate the cell type–specific contribution of *Idol* deletion to AD pathology. Here we demonstrate that neuronal, but not microglial, *Idol* deletion decreases Aβ levels. Furthermore, we performed biochemical, transcriptomic, and proteomic analyses to identify potential underlying mechanisms. Our findings indicate that the neuronal IDOL plays an important role in Aβ accumulation, gliosis, and the regulation of inhibitory neurons involved in synaptic organization via LDLR and Reelin receptors.

## 2 METHODS

### 2.1 Mice

5XFAD mice^21^ (Stock# 34840‐JAX), *Idol^f/f^* (also known as *Mylip^f/f^*)^22^ (Stock# 034728, generated by Dr. Peter Tontonoz, HHMI), *Cx3cr1‐CreERT2* ^23^ (Stock# 021160), and *Camk2a‐Cre* ^24^ (Stock# 005359) were purchased from The Jackson Laboratory. To generate microglial *Idol* KO mice, 5XFAD mice were crossed with *Idol^f/f^* and *Cx3cr1‐CreERT2* mice. Offspring were administered tamoxifen (75 mg/kg, intraperitoneally [i.p.]) once daily for 5 consecutive days at 1.5 months of age to induce microglial *Idol* deletion in 5XFAD mice (denoted as 5XFAD;MG cKO). Littermate 5XFAD controls received corn oil (vehicle). For neuronal *Idol* deletion, 5XFAD mice were crossed with *Idol^f/f^* and *Camk2a‐Cre* mice. We collected the brain tissues from 5XFAD;MG cKO, neuronal *Idol* KO 5XFAD mice (referred to as 5XFAD;Neu cKO), and 5XFAD littermate controls at 4 months of age, the age that we can reliably detect pathological changes as well as observe the expected beneficial effects without overwhelming pathology. Female mice were used in all experiments, as females exhibit more pronounced amyloid pathology in the 5XFAD model.^21^ In addition, we performed power analyses using our previously published data from 4.5‐ and 8‐month‐old 5XFAD mice to identify the cohort with the least variability.^25^, ^26^ These analyses identified that female mice showed less variability in pathological measures. Sample sizes were determined based on the statistical power calculations from our experience and other studies in the literature.^21^, ^26^, ^27^ Using a standard deviation (SD) of 150, an effect size of 60% for insoluble Aβ42 levels, a power of 0.8, and p < 0.05, we aimed to generate 12 mice per genotype. The order of animals was randomized for each experiment. The researchers were blinded during the quantification of histology samples. Figure legends contain sample sizes and statistical tests used. Animals were housed under standard conditions with ad libitum access to food and water. All procedures were approved by and conducted in accordance with the guidelines of the Institutional Animal Care and Use Committee at Indiana University (18076).

**RESEARCH IN CONTEXT**

1. **Systematic review**: The authors reviewed the literature on apolipoprotein E (*APOE*), the strongest genetic risk factor for Alzheimer's disease (AD), and its receptors implicated in disease pathogenesis. Prior research establishes that increasing APOE receptors mitigates AD pathology. However, direct pharmacological upregulation of these proteins remains challenging. Therefore, targeting endogenous negative regulators of these receptors, such as IDOL may offer new therapeutic strategies for AD.
2. **Interpretation**: We demonstrate that deletion of neuronal, but not microglial, *Idol* reduces amyloid accumulation and alters levels of brain APOE and its receptors. Our findings suggest that inhibiting neuronal IDOL may offer multiple therapeutic benefits in AD by reducing amyloid burden and gliosis while enhancing synaptic health.
3. **Future directions**: Future research should determine whether neuronal IDOL inhibition can therapeutically alter AD pathologies and cognitive outcomes and clarify its broader impact on disease progression.

### 2.2 Brain sample preparation

Mice were deeply anesthetized with Avertin (250 mg/kg, i.p.) and transcardially perfused with cold phosphate‐buffered saline (PBS). Brains were rapidly removed, and the left hemisphere was fixed in 4% paraformaldehyde at 4°C for 24 h for subsequent histological analysis. Fixed tissues were embedded in paraffin and sectioned at the Histology Core of the Indiana University School of Medicine. Five‐µm‐thick coronal sections were mounted onto microscope slides and stored at room temperature. The right hemisphere was dissected to isolate cortex and hippocampus and immediately frozen in dry ice. These samples were stored at –80°C until further biochemical experiments.

### 2.3 Protein extraction and Western blotting

Sequential protein extraction was performed on cortex and hippocampus samples using PBS, radioimmunoprecipitation assay buffer (RIPA), and 5 M guanidine buffer, supplemented with protease and phosphatase inhibitors, as we described previously.^26^ Protein concentrations in the RIPA fractions were determined using the Pierce Bicinchoninic Acid (BCA) Protein Assay kit (ThermoFisher Scientific, 23225). Protein lysates were mixed with 4X sodium dodecyl sulfate (SDS) loading dye and incubated at 95°C for 5 min. Equal amounts of protein (10 µg) were loaded onto 4%–20% TGX gels (Bio‐Rad, Cat #5671095), separated by gel electrophoresis, and transferred onto polyvinylidene difluoride (PVDF) membranes. The membranes were probed with antibodies against LDLR (MBL, JM‐3839‐100, 1:2000), APOE (Abclonal, A16344, 1:1000), APOER2 (Abcam, ab108208, 1:1000), VLDLR (R&D Systems, AF2258, 1:1000), or β‐actin (Sigma, A1978, 1:20,000). Signals were visualized using a chemiluminescence detection kit (Lumigen). Band intensities were quantified by densitometry using ImageJ software (National Institutes of Health [NIH]). Results were normalized to glyceraldehyde 3‐phosphate dehydrogenase (GAPDH) or β‐actin levels and expressed as fold‐change relative to 5XFAD.

### 2.4 Electrochemiluminescence assay for Aβ detection

For Aβ40 and Aβ42 detection, V‐PLEX Plus Aβ Peptide Panel 1 (6E10) Kit (Meso Scale Discovery, K15200E) was used. Insoluble Aβ40 and Aβ42 levels were measured in the guanidine fraction, and soluble Aβ40 and Aβ42 levels were measured in the RIPA fraction of the brain lysates following the manufacturer's instructions. Signals were measured on a MESO QuickPlex SQ 120 imager (Meso Scale Discovery).

### 2.5 Immunohistochemistry

Coronal brain sections were first deparaffinized and subjected to antigen retrieval using 1X immunohistochemistry (IHC) antigen retrieval solution (ThermoFisher, 00‐4955‐58) in a 70°C water bath. For 3,3'‐Diaminobenzidine (DAB) immunohistochemistry, slides were treated with 0.3% hydrogen peroxide, then blocked with tris‐buffered saline (TBS) containing 3% milk and 0.25% Triton‐X at room temperature for 30 min. The sections were incubated overnight at 4°C with an anti‐Aβ 82E1 antibody (IBL‐AMERICA, 10323, 1:500) in blocking solution, followed by a 1 h incubation at room temperature with a biotinylated goat anti‐mouse secondary antibody (Vector Laboratories, BA‐9200, 1:400). Antibody binding was visualized with Vectastain ABC Elite (Vector Laboratories, PK6100) and DAB peroxidase (horseradish peroxidase) substrate kits (Vector Laboratories, SK‐4100), following the manufacturer's instructions. After staining, sections were dehydrated through increasing concentrations of ethanol (50%–95%), cleared in xylene, and mounted with Permount mounting medium (Fisher Scientific, SP15100).

For fibrillar plaque detection, sections were permeabilized with 0.25% Triton X‐100 in PBS for 30 min at room temperature on a shaker and then incubated with 0.01 mM X‐34 dye (Sigma, SML1954) in a staining buffer containing 40% ethanol and 0.02N NaOH in PBS for 20 min. After rinsing with a washing buffer (40% ethanol in PBS) and PBS, coverslips were applied using Aqua‐Poly mounting medium (PolySciences Inc., 18606).

For immunofluorescence staining, sections were blocked with PBS containing 5% normal donkey and normal goat serum for 1 h at room temperature and then incubated overnight with either anti‐ionized calcium‐binding adaptor molecule 1 (IBA1) (Abcam, Ab178846, 1:1000) or anti‐glial fibrillary acidic protein (GFAP) (ThermoFisher, 13‐0300, 1:1000) antibodies in blocking solution. The following day, sections were incubated for 2 h at room temperature with Alexa Fluor 568‐conjugated goat anti‐rabbit (ThermoFisher, A11036, 1:1000) or Alexa Fluor 488‐conjugated donkey anti‐rat (Jackson ImmunoResearch, 712‐545‐150, 1:1000) secondary antibodies, and finally mounted with Aqua‐Poly/Mount medium.

### 2.6 Quantification of immunohistochemistry data

Images were captured on a digital pathology slide scanner (LEICA Biosystems, Aperio VERSA). Staining was quantified using Fiji (ImageJ, NIH) and CellProfiler 3.1.9 (Broad Institute). The average of three sections from different anatomic coordinates (150 µm distant) was used to represent plaque, IBA1+, or GFAP+ area for each mouse. The number of plaques was normalized by the total area of the analyzed region.

### 2.7 Protein preparation and liquid chromatography–mass spectrometry (LC‐MS)

Posterior cortical tissues from 5XFAD;Neu cKO and 5XFAD mice were used for proteomics analysis (N = 8/genotype). Sample processing for proteomics was done similarly to previously published work.^28^ A total of 80 µg of protein was treated with 5 mM tris(2‐carboxyethyl)phosphine and 10 mM chloroacetamide. Samples were digested with Lys‐C for 12 h at 37°C, treated with PNGase F (New England BioLabs, P0705L), and digested with Trypsin Platinum (Promega, VA9000) and quenched with trifluoroacetic acid.^29^, ^30^ The peptides were desalted with Waters Sep‐Pak Vac cartridges, dried, and resuspended in 50 mM triethylammonium bicarbonate. Fifty micrograms of peptides from each sample were labeled with 0.5 mg of TMTpro (Thermo Fisher Scientific, A44520).^30^ The labeling reaction was quenched with 0.3% hydroxylamine, multiplexed, dried, desalted, resuspended with 0.1% formic acid and 70% acetonitrile, and lyophilized. Lyophilized peptides were fractionated with Thermo UltiMate 3000 HPLC using 10 mM ammonium formate, pH 10 in water (buffer A) or 95% acetonitrile (buffer B). Fractions were collected at 1 mL/min for 110 min, interval concatenated into 48 fractions, dried, and resuspended in 80 µL of 0.1% formic acid prior to LC‐MS.^31^, ^32^, ^33^ Nano‐LC‐MS/MS analyses were performed on an EASY‐nLC HPLC system (Thermo Fisher Scientific) coupled to Orbitrap Eclipse Tribrid mass spectrometer (Thermo Fisher Scientific) with a high‐field asymmetric ion mobility spectrometry (FAIMS) Pro Interface (Thermo Fisher Scientific) at a compensation voltage of −50 V. Each fraction was loaded onto a reversed‐phase Aurora Ultimate C18 ultra‐high performance liquid chromatography (UHPLC) column (IonOpticks, AUR3‐25075C18) at 400 nL/min. Peptides were eluted from 6% to 34% with mobile phase B (80% acetonitrile with 0.1% formic acid). The mass spectrometer method was operated in positive ion mode with a 4 s cycle time data‐dependent acquisition method with advanced peak determination and Easy‐IC (internal calibrant). The data were recorded using Thermo Fisher Scientific Xcalibur 4.3 software.

### 2.8 Proteome analysis

The resulting RAW files were analyzed in Proteome Discover 2.5 (Thermo Fisher Scientific) with *Mus musculus* UniProt Reference Proteome FASTA (Proteome ID: UP000000589, downloaded on 07/2021) plus common contaminants. SEQUEST‐HT searches were conducted with a maximum number of three missed cleavages; precursor mass tolerance of 10 ppm; and a fragment mass tolerance of 0.02 Da. Static modifications used for the search were (1) carbamidomethylation on cysteine residues; and (2) TMTpro label on lysine (K) residues and the N‐termini of peptides. Dynamic modifications used for the search were TMTpro label on deamidation of asparagine; oxidation of methionine, phosphorylation on serine, threonine, or tyrosine; and acetylation, methionine loss, or acetylation with methionine loss on protein N‐termini. TMT ion intensities were normalized by total peptide amount with no scaling. Quantification methods utilized lot‐specific TMTpro isotopic impurity levels available from Thermo Fisher Scientific. The mean abundances within each genotype, fold‐change, and *p*‐values from the *t*‐test were calculated. Data shown for each protein are the mean abundance value of 5XFAD;Neu cKO samples divided by the mean abundance value of 5XFAD mean normalized abundance value ratios as the fold‐change.

### 2.9 Total RNA extraction and quantitative polymerase chain reaction (qPCR)

Total RNA was extracted from cortical tissues using TRI Reagent (MRC, TR 118). The concentration and quality of RNA were determined using the Nanodrop 2000 Spectrophotometer (Thermo Fisher). For qPCR, messenger RNAs (mRNAs) were reverse transcribed with High‐Capacity cDNA Reverse Transcription kit (Applied Biosystems). qPCR was performed in QuantStudio 3 using the default thermal cycling conditions with Power SYBR (Applied Biosystems) with the following primers: *Mylip* forward primer, CAACCAGAACACCGCCCAATA; and reverse primer, CAGCTCCTTATGCTTCGCAAC. Mouse *Gapdh* endogenous control was used as a normalization reference. Relative mRNA levels were calculated by the comparative Ct method.

### 2.10 Bulk RNA‐seq library preparation and sequencing

#### 2.10.1 Sequencing read alignment

RNA sequencing was outsourced to Lexogen Inc. (Austria). The QuantSeq 3′ mRNA‐Seq for Illumina FWD kit (version 015UG009V0252) was used, and libraries were sequenced on an Illumina NextSeq 500 using the 75 bp single‐read kit. Processing of the FastQ files was performed according to the “QuantSeq 3‘ mRNA‐Seq Integrated Data Analysis Pipelines on BlueBee Genomics Platform” user guide on the Indiana University high‐performance computing system Carbonate. All steps were performed using the parameters suggested by Lexogen in the above‐mentioned user guide, as described previously.^26^ In short, adapter sequences and the poly(A) tails were trimmed using the bbduk function in bbtools v. 38.72.^34^ FastQC v.0.11.5 was used to determine the quality of trimmed reads. The indexed mouse reference genome was generated using STAR v.2.7.3a,^35^ and we used both the mouse reference genome GRCm39 v103 and the mouse annotations files from Ensembl.^36^ STAR v.2.7.3a was used to map the trimmed reads to the indexed mouse reference genome, and samtools v1.10^37^ was used to index the bam files. Finally, the gene‐counting matrix was generated using the htseq‐count function in HTSeq v.0.12.3 with Python v.3.6.^38^

#### 2.10.2 Differential gene expression analysis

Differential gene expression analysis was performed on the resulting gene read count files using DESeq2 in R v.4.0.4.^39^ In short, a DESeq dataset was created using the DESeqDataSetFromHTSeqCount function with the design argument ∼ group (5XFAD;Neu cKO). Genes with a total read count of less than or equal to 10 were removed from the dataset. Results were plotted using the ggplot2 package.^40^

### 2.11 Single‐nuclei RNA‐seq analysis

#### 2.11.1 Single‐nuclei RNA library preparation

Nuclei from frozen posterior cortical tissue from female 5XFAD and 5XFAD;Neu cKO (N = 2/genotype) were isolated using the Singulator 100 (S2 Genomics).^41^ Briefly, frozen brain samples were added to precooled Nuclei Isolation Cartridges (S2 Genomics, 100‐063‐623) along with RNAse inhibitor (1U/µL; Roche, 03335402001) and loaded into the Singulator 100 to run on Standard Nuclei Isolation Protocol v2. The nuclei suspensions were transferred to conical tubes and pelleted at 500 g for 5 min at 4°C. Once pelleted, the supernatant was aspirated, and the pellets were resuspended in 20% Percoll (100‐253‐628; S2 Genomics) diluted in Nuclear Storage Reagent (NSR; 100‐063‐623; S2 Genomics). The samples were centrifuged at 700 g for 8 min at 4°C. The nuclei were washed with the Resuspension Buffer (2% bovine serum albumin [BSA] in NSR), spun down at 500 g for 5 min at 4°C, and resuspended in the Resuspension Buffer. The number and viability of the cells were measured using trypan blue staining and observed with an EVOS XL Core microscope. Each single‐cell mix was loaded into a Chip G and run on the Chromium Controller for GEM generation and barcoding. Sample processing and library preparation were performed according to the manufacturer's instructions using the Chromium Next GEM Single Cell 3′ v3.1 dual index kit (10X Genomics) and SPRIselect paramagnetic bead‐based chemistry (Beckman Coulter Life Sciences). The cDNA and library quality were assessed using the 2100 Bioanalyzer and a High Sensitivity DNA kit (Agilent Technologies). The final library concentration was determined using a QuBit Fluorometer and the dsDNA HS assay kit (Thermo Fisher Scientific). Sequencing was carried out on a NovaSeq 6000 (v1.5 S2; Illumina) with 28‐10‐10‐91 read setup with a targeted depth of 50,000 reads per nucleus.

#### 2.11.2 Single‐nuclei RNA sequencing data analysis

Processing of the sequencing data was performed with the cellranger pipeline (v7.1.0, 10X Genomics). The filtered feature‐cell barcode matrices (including the hashtag count matrix) generated by CellRanger were loaded into SoupX (v1.6.2)^42^ in RStudio (2024.12.0) running R (v4.4.0) and cleaned using default settings. Cleaned data were then loaded into Seurat (v5.0.3).^43^ During quality control, cells with >1% mitochondrial reads or unique reads below 200 were excluded from analysis, leaving 24,610 nuclei from the 2 5XFAD samples and 23,761 nuclei from the 2 5XFAD;Neu cKO samples. Data were normalized using SCTransform (v0.4.0), and UMAP dimensional reduction and clustering were performed using the first 30 principal components and a resolution of 0.5. Cluster annotation was performed using the Seurat FindMarkers function and was manually annotated based on marker expression in conjunction with EnrichR 3.2.^27^, ^44^, ^45^ A difference in the proportion of cells per cluster was performed using the R package Speckle (1.4.0).^46^ Differential gene expression analysis was performed using a Model‐based Analysis of Single‐cell Transcriptomics (MAST; v1.30.0)^47^ function, with regression of percent ribosomal RNA, percent mitochondrial RNA, and read depth. CellChat (v2.1.0)^48^ was used to infer inter‐cluster signaling using default package settings. Pathway and network analyses were performed using the MetaCore software.

### 2.12 Quantification and statistical analysis

Statistical analyses were performed using GraphPad Prism 10 (GraphPad Software). An unpaired Student's *t*‐test was performed for the comparison of two groups. *p*‐values less than 0.05 were considered significant. *p < 0.05, **p < 0.01, ***p < 0.001, ****p < 0.0001. Data were represented as mean ± standard error of the mean (SEM). Sample sizes and statistical analyses for each experiment were indicated in the figure legends.

## 3 RESULTS

### 3.1 Neuronal *Idol* deletion decreases Aβ accumulation

We previously demonstrated that constitutive deletion of the *Idol* gene ameliorated Aβ‐amyloidosis pathology.^20^ *Idol* is expressed ubiquitously, with the highest expression in microglia and neurons in the brain.^20^, ^49^ To investigate the cell‐type–specific role of *Idol*, we generated microglia‐ and neuron‐specific *Idol* KO mice by crossbreeding *Idol*‐floxed mice (*Idol^f/f^*) with *Cx3cr1‐CreERT2* ^23^ and *Camk2a‐Cre* ^24^ lines, respectively. We then bred these mice with the 5XFAD transgenic mouse model of Aβ‐amyloidosis to assess the contribution of microglial and neuronal *Idol* deletion to AD‐related pathologies. Because *Cx3cr1‐CreERT2* is a tamoxifen‐inducible Cre model, we administered tamoxifen to 1.5‐month‐old *5XFAD;Idol^f/f^;Cx3cr1^CreERT2/+^* mice before amyloid plaque deposition began. We collected the tissues from microglial *Idol* conditional KO (5XFAD;MG cKO) and neuronal *Idol* conditional KO (5XFAD;Neu cKO) mice when they reached 4 months of age. We confirmed the downregulation of the *Idol* gene in the brain (Figure S1).

**FIGURE 1:**
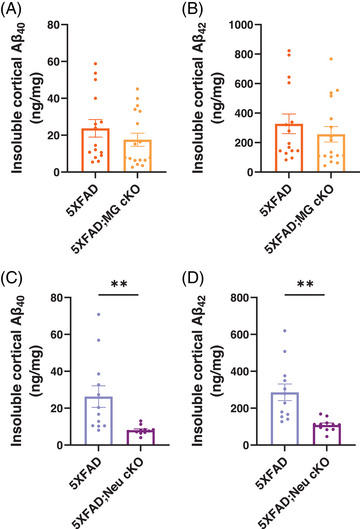
Amyloid beta (Aβ) levels decrease in the cortex of neuronal, but not microglial, *Idol* knockout mice. Insoluble Aβ40 and Aβ42 levels were measured in the guanidine fraction of 5XFAD mouse cortices using an Aβ‐electrochemiluminescence assay. (A) Insoluble Aβ40 and (B) Aβ42 levels did not change in the cortices of 4‐month‐old female microglial *Idol* knockout;5XFAD mice (5XFAD;MG cKO) compared with 5XFAD controls (N = 15–16/genotype). (C) Insoluble Aβ40 and (D) Aβ42 levels were decreased in the cortices of female neuronal *Idol* knockout;5XFAD mice (5XFAD;Neu cKO) compared with littermate 5XFAD controls (N = 11–12/genotype). The data are represented as mean ± SEM. Unpaired two‐tailed *t*‐test, **p < 0.01. (See also Figures S2 and S3).

To evaluate the impact of *Idol* deletion on Aβ accumulation, we measured the levels of Aβ proteins in the brain using an electrochemiluminescence assay, as we described previously^26^ (Figure 1). Microglial *Idol* deletion did not affect insoluble, guanidine‐extracted, Aβ40 or Aβ42 levels in the cortices of 5XFAD mice (Figure 1A,B). However, neuronal *Idol* deletion reduced insoluble Aβ40 and Aβ42 levels in the cortices of 5XFAD mice by 69% and 61%, respectively (Figure 1C,D). Soluble Aβ40 and Aβ42 levels were also decreased by 34% and 45%, respectively, in the cortices of 5XFAD;Neu cKO mice (Figure S2A,B). Because we detected changes only in 5XFAD;Neu cKO mice, we focused on this cohort to further investigate the beneficial effects of neuronal *Idol* deletion.

Next, we measured Aβ levels in the hippocampus. Similarly, insoluble Aβ40 and Aβ42 levels were decreased by 30% and 25%, respectively, in the hippocampi of 5XFAD;Neu cKO mice compared to 5XFAD mice (Figure S3A,B). Soluble Aβ40 and Aβ42 levels exhibited a trend of decrease, but these changes were not significant between the genotypes (Figure S3C,D).

**FIGURE 2:**
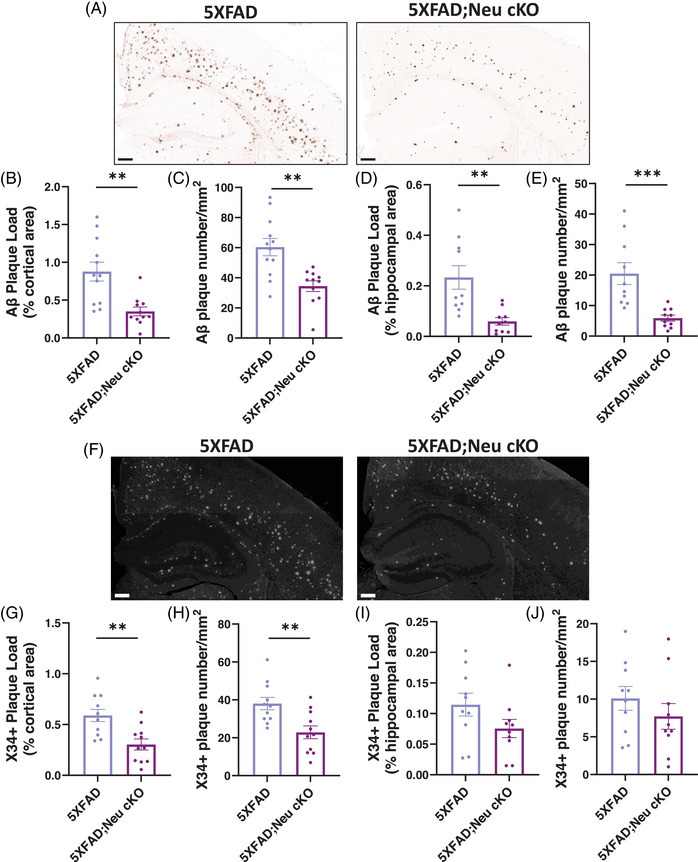
Deletion of neuronal *Idol* decreases amyloid plaques. (A) Representative images showing brain sections from 4‐month‐old 5XFAD and neuronal *Idol* knockout;5XFAD (5XFAD;Neu cKO) mice stained with an amyloid beta (Aβ)‐specific 82E1 antibody (Scale bar; 200 µm). (B) Quantification of the 82E1‐positive Aβ plaque area and (C) the number of Aβ plaques in the cortices of female mice. (D) Quantification of the 82E1‐positive Aβ plaque area and (E) the number of Aβ plaques in the hippocampus of female mice (N = 11–12/genotype). (F) Representative grayscale images of brain sections from 4‐month‐old 5XFAD and 5XFAD;Neu cKO mice stained with X34 dye that detects fibrillar plaques. The white dots represent X34+ plaques (Scale bar; 200 µm). (G) Quantification of X34+ fibrillar plaque area and (H) the number of plaques in female mouse cortices. (I) Quantification of X34+ fibrillar plaque area and (J) the number of plaques in female mouse hippocampus (N = 10–11/genotype). The data represent mean ± SEM. Unpaired two‐tailed *t*‐test, **p < 0.01, ***p < 0.001.

Misfolded Aβ peptides aggregate to form amyloid plaques. Given the reduction in Aβ peptide levels in 5XFAD;Neu cKO mice, we assessed amyloid plaque deposition by immunostaining with the Aβ‐specific 82E1 antibody that detects both diffuse and fibrillar Aβ (Figure 2A). Consistent with our biochemical analyses, we found that 5XFAD;Neu cKO mice exhibited significant decreases in both the area and the number of amyloid plaques in the cortex (Figure 2B,C). Similarly, 82E1+ amyloid plaque load and the number of plaques were decreased in the hippocampus of 5XFAD;Neu cKO mice (Figure 2D,E). To further characterize the plaque composition, we stained the sections with X34 dye that selectively binds to fibrillar sheets (Figure 2F). We detected a significant decrease in both the area and the number of fibrillar plaques in the cortices of 5XFAD;Neu cKO mice (Figure 2G,H). Although 82E1+ amyloid plaque load was decreased in the hippocampus of 5XFAD;Neu cKO mice, there was no significant difference in fibrillar amyloid plaque load (Figure 2I,J). Overall, these data demonstrate that neuronal *Idol* deletion ameliorates Aβ deposition in 5XFAD mice.

### 3.2 Gliosis decreases in neuronal *Idol* knockout mice

Gliosis is another pathological hallmark of AD, emerging at very early stages of the disease.^50^, ^51^ Although the causal link between Aβ accumulation and gliosis remains unclear, amyloid plaque formation is typically accompanied by gliosis. Numerous studies have identified changes in the number, morphology, and transcriptome of astrocytes and microglia at different stages of AD, underscoring their role in disease progression.^50^, ^51^, ^52^

**FIGURE 3:**
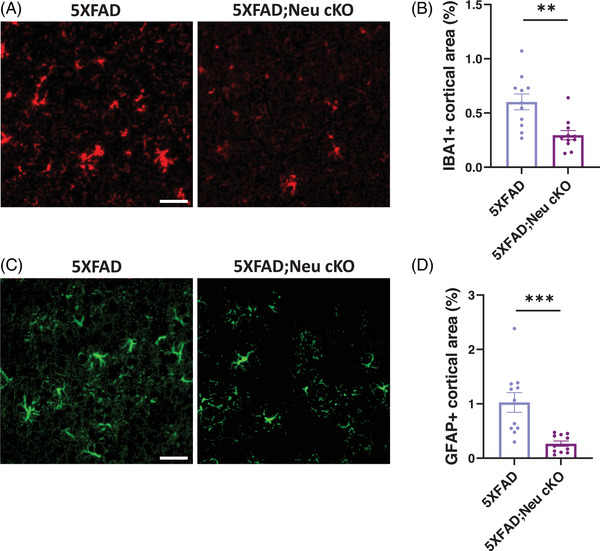
Deletion of neuronal *Idol* decreases gliosis. (A) Representative images showing brain sections stained with the myeloid marker ionized calcium‐binding adaptor molecule 1 (IBA1) antibody (red) (Scale bar; 50 µm). (B) The percentage of the area covered by IBA1 staining was quantified in the cortices of female mice. The area covered by IBA1+ cells was significantly decreased in neuronal *Idol* knockout;5XFAD (5XFAD;Neu cKO) mice compared with 5XFAD. (C) Representative images showing brain sections stained with the astrocyte marker glial fibrillary acidic protein (GFAP) antibody (green) (Scale bar; 50 µm). (D) The percentage of the area covered by GFAP was significantly decreased in 5XFAD;Neu cKO mice compared with 5XFAD (N = 11/genotype). The data represent mean ± SEM. Unpaired two‐tailed *t*‐test, **p < 0.01, ***p < 0.001.

Given the marked reduction in the amyloid plaque load in 5XFAD;Neu cKO mice, we performed immunostaining for ionized calcium‐binding adaptor molecule 1 (IBA1) and glial fibrillary acidic protein (GFAP) to label microglia and astrocytes, respectively (Figure 3). 5XFAD;Neu cKO mice exhibited a significant reduction in the IBA1+ area, indicating decreased microgliosis (Figure 3A,B). In addition, astrogliosis was decreased in 5XFAD;Neu cKO mice, as evidenced by a reduced GFAP+ area in the brain (Figure 3C,D). These findings demonstrate that neuronal *Idol* deletion decreases gliosis, suggesting reduced neuroinflammation in these mice.

### 3.3 Neuronal *Idol* deletion affects the APOE‐LDLR pathway

**FIGURE 4:**
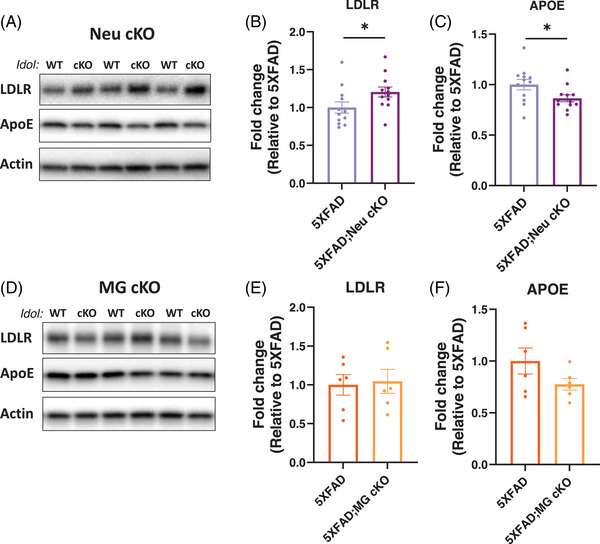
Deletion of neuronal, but not microglial, *Idol* increases LDLR levels and decreases APOE levels in the brain. Protein levels in the RIPA fraction of female mouse cortices were determined by Western blot. (A) Representative images of blots from 5XFAD and 5XFAD;Neu cKO mice. Quantification of (B) LDLR and (C) APOE immunoblots (N = 12/genotype). (D) Representative images of blots from 5XFAD and 5XFAD;MG cKO mice. Quantification of (E) LDLR and (F) APOE immunoblots. The data were normalized by β‐actin and expressed as the fold‐change relative to the 5XFAD (N = 6/genotype). The data represent mean ± SEM. Unpaired two‐tailed *t*‐test, *p < 0.05. APOE, apolipoprotein E; cKO, conditional knockout; LDLR, low‐density lipoprotein receptor; MG cKO, microglial *Idol* knockout; Neu cKO, neuronal *Idol* knockout; RIPA, radioimmunoprecipitation assay; WT, wild‐type.

Since IDOL targets LDLR for its degradation, we assessed LDLR levels in the brains of 5XFAD;MG cKO and 5XFAD;Neu cKO mice using Western blot analysis (Figure 4). As LDLR is an APOE receptor responsible for APOE uptake, we also measured the levels of APOE in the brain. Neuronal *Idol* deletion significantly increased LDLR and decreased APOE levels (Figure 4A–C). Surprisingly, however, 5XFAD;MG cKO mice showed no significant changes in either protein (Figure 4D–F). Given that Aβ levels did not change in 5XFAD;MG cKO mice, these important negative findings suggest that the beneficial effects of *Idol* deletion on amyloid pathology may depend on brain LDLR and APOE levels. Although neuronal *Idol* deletion was sufficient to increase total brain LDLR levels, microglial *Idol* deletion was not. Consequently, the levels of APOE and Aβ were decreased only in 5XFAD;Neu cKO mice, not in 5XFAD;MG cKO mice. These results highlight the critical role of the LDLR‐APOE axis in Aβ accumulation and its regulation by neuronal IDOL.

**FIGURE 5:**
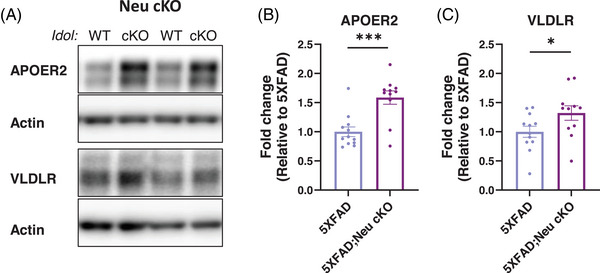
Levels of Reelin receptors are increased by neuronal *Idol* deletion. Protein levels of Reelin receptors were determined in the RIPA fraction of female mouse cortices by Western blot. (A) Representative images of blots from 5XFAD and 5XFAD;Neu cKO mice. Quantification of (B) APOER2 and (C) VLDLR immunoblots. The data were normalized by β‐actin and expressed as the fold‐change relative to the 5XFAD (N = 11–12/genotype). The data represent mean ± SEM. Unpaired two‐tailed *t*‐test, *p < 0.05, ***p < 0.001. APOER2, apolipoprotein E receptor 2; cKO, conditional knockout; Neu cKO, neuronal *Idol* knockout; RIPA, radioimmunoprecipitation assay; VLDLR, very low‐density lipoprotein receptor; WT, wild type.

In addition to LDLR, IDOL also targets other APOE receptors, APOER2 and VLDLR, which are key mediators of Reelin signaling.^53^ The Reelin pathway plays an essential role in synaptic functions.^54^ To assess whether neuronal *Idol* deletion affects these Reelin receptors, we measured their protein levels by Western blot (Figure 5A). Similar to LDLR, we detected a significant increase in APOER2 and VLDLR levels in 5XFAD;Neu cKO mice, indicating that neuronal IDOL regulates multiple APOE receptors important for AD pathogenesis (Figure 5B,C).

### 3.4 Lipid metabolism–related pathways are altered due to neuronal *Idol* deletion

**FIGURE 6:**
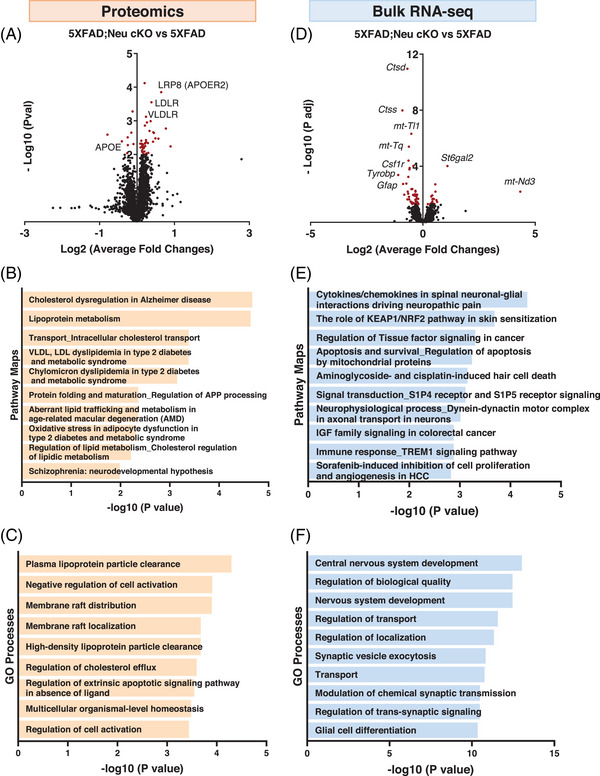
The effect of neuronal *Idol* deletion on the brain proteome and transcriptome. (A–C) Mass spectrometry was performed on the cortices of neuronal *Idol* knockout;5XFAD (5XFAD;Neu cKO) and 5XFAD mice (N = 8/genotype). (A) Volcano plot showing the differential expression of proteins in 5XFAD;Neu cKO mice compared with 5XFAD mice. Red dots represent significantly up‐ or downregulated proteins in 5XFAD;Neu cKO mice compared with 5XFAD mice (*p* < 0.01). (B) Pathway analysis of differentially expressed proteins (DEPs) and (C) Gene ontology analysis of DEPs using the MetaCore software. (See also Tables S1–S3.) (D–F) Bulk RNA‐seq was performed on the cortices of 5XFAD;Neu cKO and 5XFAD mice (N = 5/genotype). (D) Volcano plot showing the differentially expressed genes (DEGs) in 5XFAD;Neu cKO mice compared with 5XFAD mice. The x‐axis shows the fold‐change, and the y‐axis shows the statistical significance level expressed as the −log10 P adjusted. The red dots represent genes significantly (p adj < 0.05) up‐ or downregulated in 5XFAD;Neu cKO mice compared with 5XFAD mice. (E) Pathway and (F) Gene ontology (GO) analyses were performed using the MetaCore. (See also Figure S4 and Table S3‐S6.)

To gain further insight into the potential mechanisms by which neuronal *Idol* deletion reduces Aβ accumulation, we performed deep mass spectrometry–based proteomics on the cortices of 5XFAD;Neu cKO mice (Figure 6). We were able to detect 11,829 proteins with extensive liquid chromatography fractionation. We used *p* < 0.01 as a cutoff to determine the differentially expressed proteins (DEPs) (Table S1). Among the DEPs, LDLR was significantly upregulated and APOE was downregulated, corroborating our Western blot data shown in Figure 4 (Figure 6A). VLDLR and APOER2 (LRP8), known IDOL targets,^53^ were similarly upregulated in 5XFAD;Neu cKO mice (Figure 6A), as confirmed by Western blot (Figure 5).

To better understand the biological processes regulated by DEPs in 5XFAD;Neu cKO mice, we performed pathway analysis using the MetaCore software. These analyses revealed enrichment of lipid metabolism and cholesterol transport pathways, with “Cholesterol dysregulation in Alzheimer's disease” being the top pathway (Figure 6B and Table S2). Because LDLR mediates the uptake of APOE‐bound lipid particles and Aβ, changes in the LDLR level may alter the cellular lipid content and contribute to the reduced Aβ levels in 5XFAD;Neu cKO mice. Gene ontology (GO) analysis further revealed that “lipoprotein particle clearance” and “membrane raft distribution” were significantly altered processes in 5XFAD;Neu cKO mice, supporting the role of neuronal IDOL in brain lipid metabolism (Figure 6C and Table S3).

### 3.5 Transcriptomic changes in neuronal *Idol* knockout mice

IDOL induces post‐translational modifications by ubiquitinating its substrates,^19^, ^53^ but neuronal *Idol* deletion may also result in transcriptomic changes. To investigate this, we performed bulk RNA sequencing (RNA‐seq) on the cortices of 5XFAD;Neu cKO mice (Figure 6D). We detected downregulation of proteolytic enzymes, such as cathepsin D (*Ctsd*) and cathepsin S (*Ctss*), as well as immune‐related genes, including colony‐stimulating factor 1 receptor (*Csf1r*), TYRO protein tyrosine kinase‐binding protein (*Tyrobp*), and *Gfap* (Figure 6D and Table S4). Pathway analysis of differentially expressed genes (DEGs, p‐adj < 0.05) revealed enrichment of the immune response and apoptosis‐related pathways (Figure 6E and Table S5). Furthermore, GO analysis revealed enrichment of biological processes related to transport and synaptic transmission in 5XFAD;Neu cKO mice (Figure 6F and Table S6).

We further investigated these changes by performing integrative analyses with DEGs from bulk RNAseq and DEPs from the proteomics data (Figure S4). This approach provided a more detailed view of molecular alterations in 5XFAD;Neu cKO mice compared with the individual analyses. Pathway analysis identified distinct patterns across datasets, but also jointly highlighted the “Protein folding and maturation,” “Aberrant lipid trafficking,” and “Cholesterol dysregulation in Alzheimer's disease” pathways (Figure S4A). These findings further strengthen the role of neuronal IDOL in regulating lipid metabolism and protein accumulation in AD. Network analysis also identified divergent patterns across the two datasets (Figure S4B). Although translation, cell adhesion, and inflammation‐related pathways were driven mainly by transcriptomic alterations, the enrichment of proteolysis, cell cycle, and signal transduction pathways was primarily influenced by proteomic changes (Figure S4B). These differences between transcriptomic and proteomic datasets may reflect post‐transcriptional regulatory mechanisms.

**FIGURE 7:**
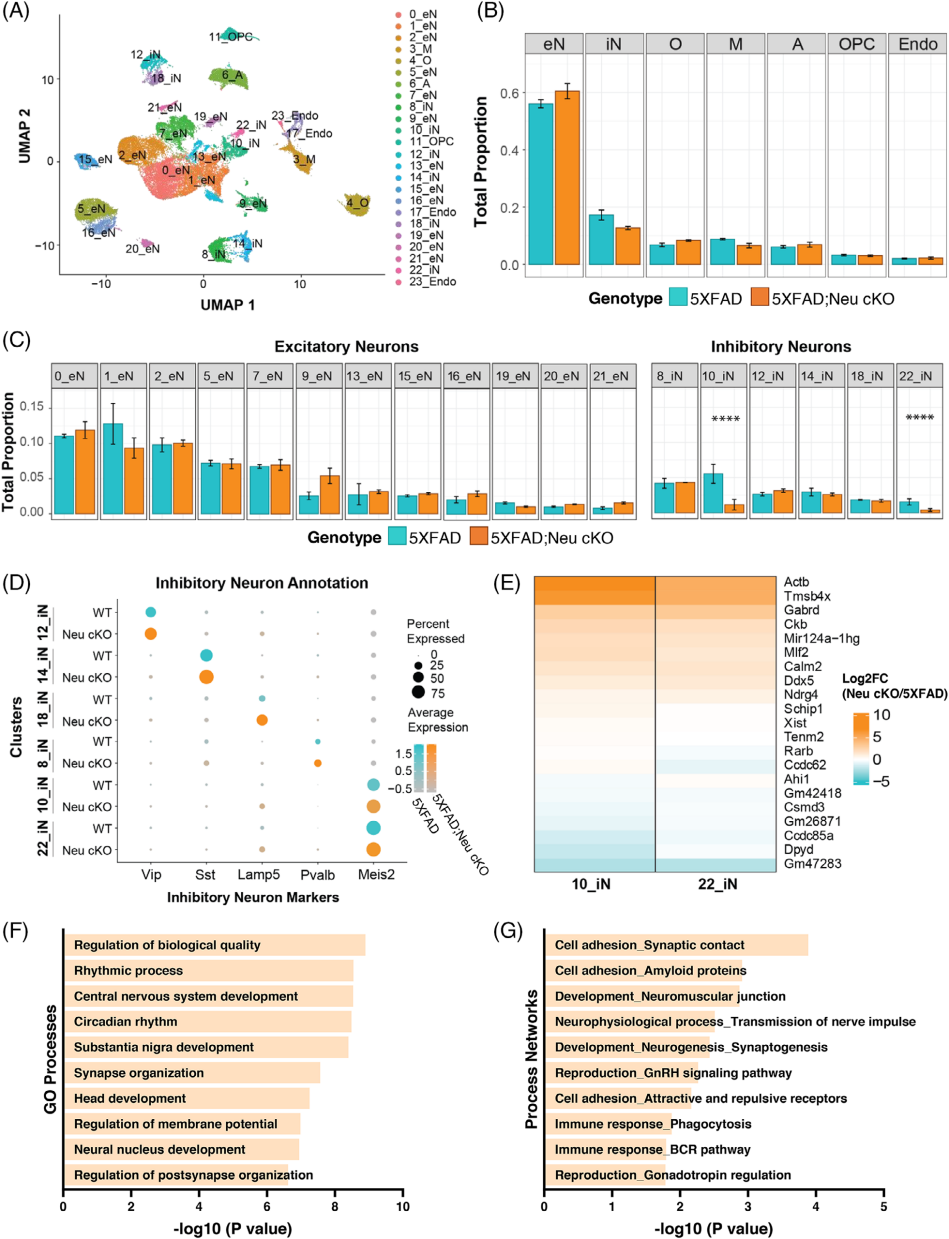
snRNA‐seq identifies changes in inhibitory neuron populations in neuronal *Idol* knockout mice. snRNA‐seq was performed on the cortices of 5XFAD;Neu cKO and 5XFAD mice. (A) Uniform Manifold Approximation and Projection (UMAP) dimensionality reduction plot showing 24 clusters of cells derived from 4‐month‐old female 5XFAD;Neu cKO and 5XFAD mice (N = 24,610 nuclei from 2 5XFAD, N = 23,761 nuclei from 2 5XFAD;Neu cKO mice). The cell types are displayed after each cluster number in the legend. eN = excitatory neurons, iN = inhibitory neurons, O = oligodendrocytes, M = microglia, A = astrocytes, OPC = oligodendrocyte progenitor cells, Endo = endothelial cells. (B) The proportion of the main cell types in each genotype. (C) The distribution of each cluster in excitatory and inhibitory neurons was compared between the genotypes. The proportions of clusters 10_iN and 22_iN were significantly decreased in 5XFAD;Neu cKO mice. ****FDR < 0.0001. (D) Inhibitory neuron clusters were identified using inhibitory neuron markers (*Vip, Sst, Lamp5, Pvalb, Meis2*) and compared between the genotypes. The size of each dot indicates the proportion of cells within a cluster expressing the gene, whereas the color intensity reflects the average scaled expression level across clusters. The significantly decreased Cluster 10 and 22 inhibitory neurons were identified as *Meis2+* neurons. (E) Heatmap demonstrating the top 20 differentially expressed genes (DEGs) in Clusters 10 and 22. The log2 fold‐change of gene expression is shown in each cluster. The upregulated genes in 5XFAD;Neu cKO are shown in orange, and downregulated genes are shown in blue. (F) Gene ontology analysis of DEGs in Clusters 10 and 22, and (G) Network analysis of DEGs using the MetaCore software. (See also Figures S5–S7; Tables S7, S8, S23, and S27–S30.). *Lamp5*, lysosomal‐associated membrane protein family member 5; *Meis2*, meis homeobox 2; Neu cKO, neuronal *Idol* knockout; *Pvalb*, parvalbumin; snRNA‐seq, single nuclei RNA‐sequencing; *Sst*, somatostatin; *Vip*, vasoactive intestinal polypeptide; WT, wild type for *Idol*.

Bulk RNA‐seq provides useful insights; however, it lacks cell‐type specificity. To investigate the impact of neuronal *Idol* deletion across cell types, we performed single‐nuclei RNA‐sequencing (snRNA‐seq)^41^ on the cortices of 5XFAD;Neu cKO mice (Figure 7). Unsupervised clustering identified 24 distinct clusters across the samples (Figure 7A). These clusters were annotated based on the expression of known cell type–specific markers: excitatory neurons (eN) (Clusters 0–2, 5, 7, 9, 13, 15, 16, 19–21), inhibitory neurons (iN) (Clusters 8, 10, 12, 14, 18, and 22), microglia (M) (Cluster 3), oligodendrocytes (O) (Cluster 4), astrocytes (A) (Cluster 6), oligodendrocyte progenitor cells (OPC) (Cluster 11), and endothelial cells (Endo) (Clusters 17 and 23) (Figure 7A and Table S7). Neuronal *Idol* deletion did not alter the proportions of major cell types in the brain (Figure 7B and Table S8). To investigate whether neuronal *Idol* deletion affected their transcriptome, we performed DEG analyses within each cell type (Figures S5 and S6). Even though snRNA‐seq analysis was performed on a modest sample size (N = 2 mice/genotype), given the high yield of our single nuclei isolation protocol, we were able to detect transcriptomic changes across multiple cell types. Inhibitory and excitatory neurons exhibited marked differences in gene expression between 5XFAD;Neu cKO mice and 5XFAD controls (Figure S5 and Tables S9–S12). Pathway analyses demonstrated that DEGs in inhibitory neurons were primarily involved in protein folding and synaptic processes (Figure S5A,B and Tables S9 and S10). Excitatory neuron DEGs were similarly enriched in synapse‐related pathways but also showed enrichment in distinct pathways, such as calcium‐mediated signaling and G‐protein signaling (Figure S5C,D and Tables S11 and S12). Notably, neuronal *Idol* deletion significantly altered the transcriptome not only within neuronal subpopulations but also in other brain cell types, including microglia, astrocytes, oligodendrocytes, oligodendrocyte precursor cells, and endothelial cells (Figure S6 and Tables S13–S22). Although these findings provided valuable initial insight into the molecular consequences of neuronal *Idol* deletion, larger sample sizes will be required to more comprehensively capture transcriptomic alterations and account for biological variability.

Because we identified multiple clusters in neuronal populations, we performed subcluster analyses in these cell types (Figure 7C). There was no significant difference in the proportion of excitatory neuron clusters, but neuronal *Idol* deletion significantly reduced the proportion of cells in inhibitory neuron Clusters 10 and 22 (Figure 7C and Table S23). To further characterize these clusters, we focused our analysis on inhibitory neuron clusters. We identified five distinct inhibitory neuron subtypes: vasoactive intestinal polypeptide (*Vip*), somatostatin (*Sst*), lysosomal‐associated membrane protein family member 5 (*Lamp5*), Parvalbumin (*Pvalb*), and Meis Homeobox 2 (*Meis2*)–positive inhibitory neurons (Figure 7D). Interestingly, both Clusters 10 and 22, the significantly reduced clusters in 5XFAD;Neu cKO mice (Figure 7C), were identified as *Meis2+* inhibitory neurons (Figure 7D). To further investigate the *Meis2+* inhibitory neurons, we compared the expression of the genes in these clusters (10 and 22) with other inhibitory neuron clusters. We identified 425 downregulated and 388 upregulated genes unique to *Meis2+* inhibitory neurons compared to other inhibitory neurons (Figure S7A and Table S24). Pathway analysis of these common DEGs highlighted enrichment in “NMDA‐independent presynaptic long‐term potentiation,” “Regulation of intrinsic membrane properties and excitability,” and “G‐protein signaling_RhoA activation” (Figure S7B and Table S25). GO analysis further revealed enrichment of synapse‐related biological processes, such as “synaptic transmission” and “regulation of trans‐synaptic signaling,” in Clusters 10 and 22 (Figure S7C and Table S26).

Given the significant reduction in the proportion of Clusters 10 and 22, we performed DEG analysis within these clusters to assess the impact of neuronal *Idol* deletion (Table S27 and S28). Because Cluster 22 had relatively few DEGs and was also identified as *Meis2+*, similar to Cluster 10 neurons, we combined the DEGs from both clusters for these analyses. The top DEGs largely showed the same directionality in Clusters 10 and 22, strengthening our decision to combine the analyses (Figure 7E). Beta‐actin (*Actb*), thymosin beta 4 X‐linked (*Tmsb4x*), and GABA‐A receptor subunit delta (*Gabrd*) were among the top DEGs (Figure 7E). To gain insight into the biological processes regulated by the DEGs, we performed pathway analysis. GO analysis identified enrichment of synapse organization and the regulation of membrane potential (Figure 7F and Table S29). Furthermore, network analysis revealed “Cell adhesion_Synaptic contact” and “Cell adhesion_Amyloid proteins” as the top process networks (Figure 7G and Table S30). These findings suggest that neuronal IDOL may contribute to AD, not only by affecting Aβ accumulation but also by modulating synaptic functions.

**FIGURE 8:**
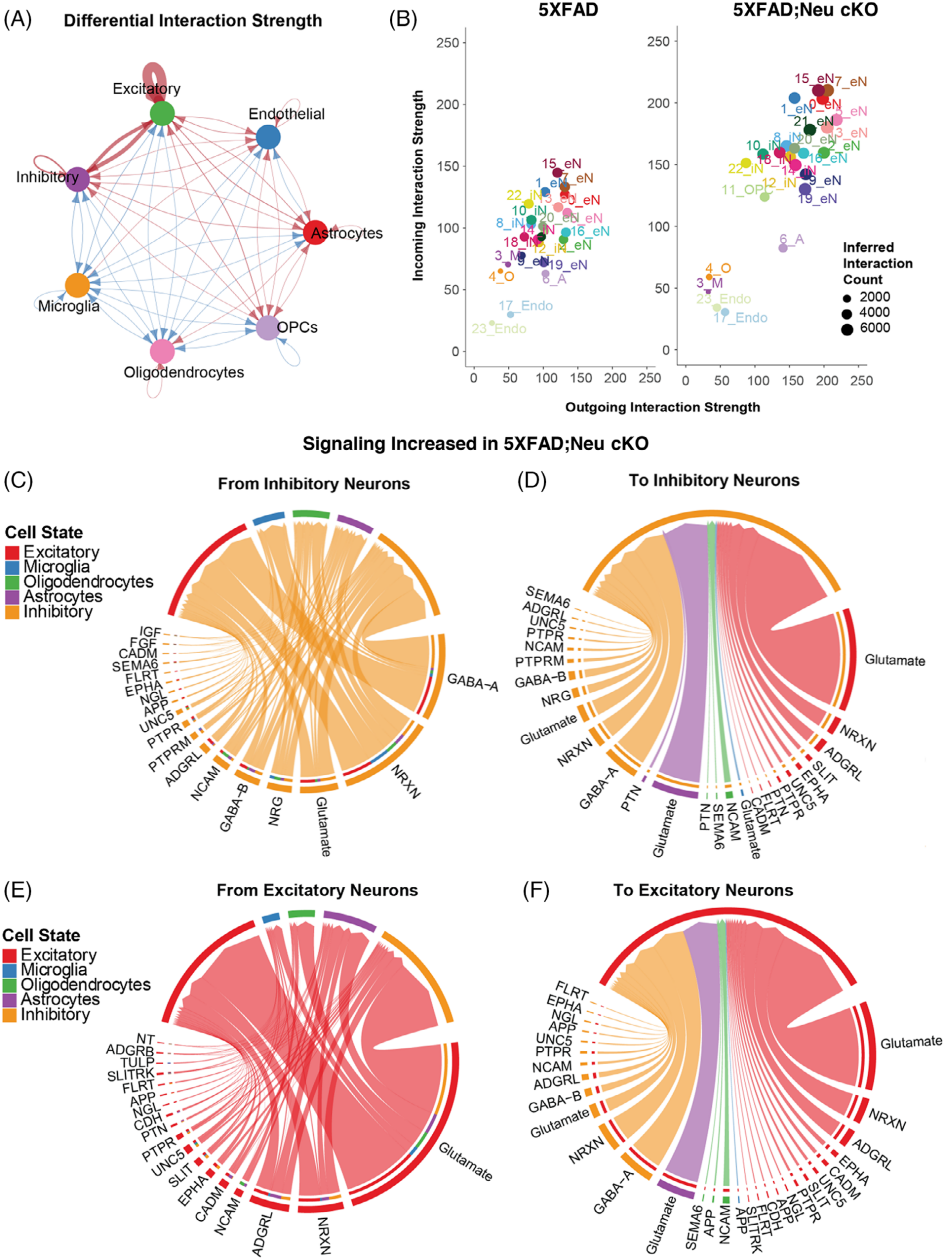
Increased neuronal signaling in neuronal *Idol* knockout mice. CellChat analysis was performed on the single nuclei RNA‐seq (snRNA‐seq) data from 5XFAD;Neu cKO and 5XFAD mice. (A) Chord plot showing the changes in interactions between the main cell types in the brains of neuronal *Idol* knockout;5XFAD (5XFAD;Neu cKO) mice compared with 5XFAD mice. Red lines indicate increased signaling between cell types, and blue lines indicate decreased signaling between cell types. The thickness of the lines represents the interaction strength. (B) The predicted interaction strengths in the cell clusters identified in 5XFAD (left panel) and 5XFAD;Neu cKO (right panel) mouse brains. The x‐axis shows the interaction strength of outgoing signals, and the y‐axis shows the interaction strength of incoming signals. Both incoming and outgoing signal strengths were increased in most cell clusters in the 5XFAD;Neu cKO mouse brain, compared to the 5XFAD mouse brain, except for microglia and oligodendrocytes. (C) Chord plot showing increased signaling from inhibitory neurons to other cell types in the 5XFAD;Neu cKO mouse brain. (D) Chord plot showing increased signaling to inhibitory neurons from other cell types in the 5XFAD;Neu cKO mouse brain. (E) Chord plot showing increased signaling from excitatory neurons to other cell types in the 5XFAD;Neu cKO mouse brain. (F) Chord plot showing increased signaling to excitatory neurons from other cell types in the 5XFAD; Neu cKO mouse brain. (See also Table S31.)

To assess the impact of neuronal *Idol* deletion on intercellular communication, we performed CellChat analysis. CellChat infers communication networks between cell groups based on the expression of ligands, receptors, and cofactors.^48^ We analyzed communication networks across clusters as well as those annotated by cell type. These analyses revealed an overall increase in cell‐to‐cell communication among most cell types, particularly between and within inhibitory and excitatory neurons in 5XFAD;Neu cKO mice (Figure 8A,B). In contrast, microglia and oligodendrocytes exhibited reduced incoming and outgoing interactions in 5XFAD;Neu cKO mice (Figure 8A,B).

Because the most prominent changes were observed in inhibitory and excitatory neurons, we further investigated their signaling networks. Signaling from inhibitory to excitatory neurons was markedly increased in 5XFAD;Neu cKO mice, driven primarily by the neurexin and GABA‐A pathways (Figure 8C). Neurexins, presynaptic adhesion molecules, are critical for synaptic transmission.^55^ In addition, neurexins can bind to Aβ and amyloid precursor protein (APP), thereby preventing Aβ‐induced synaptic pathology and mediating the synaptogenic activity of APP.^56^, ^57^, ^58^ Increased neurexin signaling may contribute to the enrichment of biological processes and networks such as “synapse organization,” “cell adhesion_synaptic contact,” and “cell adhesion_amyloid proteins” in 5XFAD;Neu cKO mice (Figure 7F,G). Furthermore, we identified increased signaling from excitatory neurons to inhibitory neurons, especially through the neurexin and glutamate pathways (Figure 8D and Table S31). Interestingly, there was also an increase in glutamate signaling from astrocytes, the main LDLR‐expressing cells, to inhibitory neurons (Figure 8D).

Given the increased cell‐to‐cell communication in excitatory neurons (Figure 8A), we also investigated the details of these interactions and identified increased glutamate and neurexin signaling from excitatory neurons (Figure 8E). Similar to the inhibitory neurons, there was an increase in glutamate and neurexin signaling from excitatory neurons to themselves (Figure 8F). The relative enrichment of *Apoer2 (Lrp8)* and *Vldlr* in excitatory and inhibitory neurons compared to *Ldlr* (Figure S8) suggests that increased signaling in these neurons may be related to elevated APOER2 and VLDLR levels in 5XFAD;Neu cKO mice. Collectively, these findings suggest that neuronal *Idol* deletion enhances cellular interactions, except in microglia and oligodendrocytes.

## 4 DISCUSSION

In this study, we investigated the cell type–specific role of IDOL, an E3 ubiquitin ligase that mediates the ubiquitination and degradation of LDLR, in Aβ‐amyloidosis pathology. LDLR is one of the major APOE receptors in the brain and facilitates the endocytosis of APOE‐bound lipoprotein particles.^59^ Because *APOE* was identified as a genetic risk factor for AD, numerous studies have sought to elucidate how APOE contributes to AD pathogenesis. Notably, deletion of *Apoe* reduces amyloid deposition in transgenic mouse models of AD.^3^, ^4^ Because LDLR is a major endocytic APOE receptor,^59^ it was hypothesized that modulating LDLR levels could influence brain APOE levels. Indeed, deletion of *Ldlr* increased APOE levels in the brain.^59^, ^60^, ^61^ Of note, the overexpression of *Ldlr* significantly reduced APOE and Aβ levels in Aβ‐amyloidosis mouse models.^16^, ^18^ These findings underscore the critical role of LDLR in regulating Aβ accumulation and position it as a potential therapeutic target for AD. Because IDOL promotes the degradation of LDLR, we hypothesized that inhibiting IDOL could be a promising approach to increase endogenous LDLR levels and, consequently, reduce Aβ deposition. Indeed, our previous work using a whole‐body *Idol* KO mouse model demonstrated an increase in brain LDLR levels with a significant decrease in Aβ accumulation.^20^ In addition, the inhibition of *Idol* with an antisense oligonucleotide was reported to reduce the amyloid burden in an *APP* transgenic mouse model.^62^ Because *Idol* is expressed primarily in microglia and neurons in the brain, we examined its role in these cell types and its contribution to amyloid pathology.

Since APOE regulates Aβ clearance via LDL receptors and microglia are critical for Aβ clearance,^15^ we first focused on the role of IDOL in microglia. Previously demonstrated that *Idol* knockdown increased Aβ uptake in microglia in vitro.^20^ This effect was abolished with *Ldlr* knockdown,^20^ suggesting that LDLR mediates the impact of *Idol* inhibition on microglial Aβ uptake. Moreover, the inhibition of IDOL with an antisense oligonucleotide induced transcriptomic changes mainly in microglia populations, as detected by single‐cell RNA‐seq.^62^ Based on the enrichment of DEGs in lysosomal pathways, it was suggested that IDOL inhibition might enhance microglial phagocytic functions.^62^ However, further functional studies are necessary to establish causality and to address potential technical biases introduced by single‐cell isolation methods that enrich glial cells. To determine whether microglial *Idol* deletion decreases Aβ accumulation in vivo, we generated 5XFAD;MG cKO mice. Surprisingly, Aβ levels did not change in the brains of 5XFAD;MG cKO mice (Figure 1). While IDOL may regulate microglial Aβ clearance, its ubiquitous expression implies that additional mechanisms may contribute to the amyloid reduction observed in *Idol* constitutive KO mice.^20^ Indeed, our data demonstrate that the deletion of microglial *Idol* alone is not sufficient to decrease amyloid deposition, indicating the involvement of other mechanisms.

To investigate the contribution of neuronal IDOL to amyloid deposition, we generated neuronal *Idol* KO mice and crossbred them with 5XFAD mice (5XFAD;Neu cKO). Deletion of neuronal *Idol* markedly decreased Aβ levels and amyloid plaques (Figures 1 and 2). To explore the underlying mechanisms, we assessed LDLR and APOE levels in the brains of 5XFAD;Neu cKO and 5XFAD;MG cKO mice. Surprisingly, only neuronal *Idol* deletion increased LDLR and decreased APOE levels, whereas microglial *Idol* deletion had no effect on either (Figure 4). Given that only 5XFAD;Neu cKO mice showed reduced amyloid deposition, these findings suggest that the neuronal LDLR‐APOE axis may play an important role in Aβ accumulation.

As *APOE* ε4 is the strongest genetic risk factor for AD, many studies have focused on understanding how different *APOE* isoforms influence disease risk. These studies have discovered multiple mechanisms by which *APOE ε4* affects various cell types, contributing to AD‐related pathologies.^63^, ^64^ One key difference among *APOE* isoforms is their ability to regulate lipid metabolism and cholesterol efflux. While *APOE2* is the most efficient at promoting cholesterol efflux, *APOE4* is the least efficient, correlating inversely with AD risk.^65^, ^66^ Biochemical studies have also identified isoform‐specific differences in LDLR binding, with APOE2 exhibiting a lower binding capacity than APOE4 does.^67^, ^68^, ^69^ A recent study explored how these binding differences may affect lipid metabolism and tau pathology.^70^ Cells treated with lipidated‐APOE2 exhibited decreased APOE‐LDLR binding and reduced uptake of lipidated‐APOE, thereby preventing excessive lipid burden and lysosomal pathology compared to APOE3 and APOE4.^70^ In a tauopathy model, *APOE4* mice exhibited the greatest lipid peroxidation, whereas *APOE2* mice had the least. This difference was suggested to result from APOE‐LDLR interactions.^70^ Although this study provides a novel mechanism linking APOE‐LDLR interaction to lysosomal pathology and suggests that reducing LDLR may be beneficial, these effects may be context dependent. For example, *APOE2* mice exhibited more tau pathology, compared to *APOE3* and *APOE4* mice, and behavioral abnormalities in a tauopathy model.^71^ Furthermore, the *APOE* ε2 allele was associated with increased tau pathology in the brains of human progressive supranuclear palsy, a primary tauopathy.^71^ Similarly, *APOE2* exacerbated TDP‐43 proteinopathy,^72^ a pathology that is seen in 57% of patients with AD.^73^ As AD involves multiple pathologies, including amyloid, tau, and lysosomal pathologies, future studies should consider these pathologies together to better understand the net effect of APOE‐LDLR interaction on AD. Given the critical role of LDLR in Aβ clearance, the benefits of increasing LDLR may outweigh the overall management of AD. It is notable that overexpression of *Ldlr* also reduced tau pathology and neurodegeneration in a tauopathy mouse model,^74^ further supporting the beneficial effects of increasing LDLR in AD.

Although the impact of *APOE* isoforms on AD is well established, modulating APOE levels also affects disease pathology. For example, in a tauopathy mouse model, *Apoe* KO mice exhibited less tau‐mediated neurodegeneration compared to those expressing *APOE3* or *APOE4*.^75^ Other studies have demonstrated that deleting one copy of human *APOE* ε4 or *APOE* ε3 significantly reduces APOE levels and amyloid pathology in mouse models.^5^, ^6^ Taken together, these findings suggest that reducing APOE levels, regardless of isoform, may be beneficial in AD. Indeed, a recent human genetics study supported this notion by identifying *APOE* loss‐of‐function mutations associated with resistance to AD, independent of the *APOE* isoform.^10^ Losing one copy of either *APOE* ε3 or *APOE ε4* delayed or prevented symptom onset.^10^ Collectively, these studies provide compelling evidence that lowering APOE levels may be a promising therapeutic strategy for AD.^11^ Therefore, IDOL inhibition may be a viable approach, as it regulates LDLR‐mediated APOE uptake.

Although the beneficial effects observed in 5XFAD;Neu cKO mice may be due to decreased APOE levels (Figure 4C), this is likely not the only mechanism. Indeed, LDLR has been demonstrated to regulate Aβ uptake and clearance even in the absence of APOE, indicating APOE‐independent effects.^76^ Furthermore, both *Ldlr* KO and *Ldlr‐Apoe* double KO 5XFAD mice showed reduced astrogliosis and microgliosis, whereas *Apoe* KO mice did not.^61^ These results suggest that LDLR may also regulate neuroinflammation independently of APOE. In addition, LDLR‐independent mechanisms may contribute to the phenotype observed in 5XFAD;Neu cKO mice. VLDLR and APOER2, other LDL receptor family members, are also the targets of IDOL.^53^ Our proteomics data support this finding, as VLDLR and APOER2 levels were increased in the brains of 5XFAD;Neu cKO mice (Figure 6). VLDLR and APOER2 mediate Reelin signaling, which is essential for neuronal migration during brain development.^77^ Because we used the Camk2a‐Cre mouse model that initiates Cre‐loxP recombination postnatally, not during the embryonic stage, any potential developmental defects or compensatory responses were avoided. In the adult brain, Reelin signaling is important for memory and synaptic plasticity, as deletion of either *Reelin (Reln)* or its receptors, *Vldlr* and *Apoer2*, impairs hippocampal synaptic transmission.^54^, ^78^ Moreover, Reelin signaling has been demonstrated to mitigate Aβ‐mediated synaptic impairment and tau phosphorylation.^79^, ^80^ Therefore, the increase in VLDLR and APOER2 levels following neuronal *Idol* deletion may also contribute to the beneficial effects observed in 5XFAD;Neu cKO mice. Given that *Reln* is expressed in inhibitory neurons in the adult brain^81^, ^82^, ^83^ and our snRNA‐seq analysis identified the most significant changes in inhibitory neuron populations in 5XFAD;Neu cKO mice (Figure 7), VLDLR and APOER2 likely play a role in the observed phenotypes. The enrichment of these receptors in inhibitory neurons compared to LDLR also strengthens this hypothesis (Figure S8).

In addition to reducing Aβ pathology, inhibiting IDOL could enhance the neuroprotective effects of Reelin. Recently, a rare gain‐of‐function variant in *RELN* was identified in a patient with an autosomal dominant AD‐causing mutation, and it was associated with resilience against cognitive impairment.^84^ The individual developed mild cognitive impairment approximately two decades later than the typical onset for carriers of this autosomal dominant AD mutation and exhibited relatively low tau pathology. Biochemical and functional studies revealed that the *RELN* variant enhances Reelin signaling and limits tau pathology, which may underlie the observed cognitive resilience. Considering these studies, our findings suggest that IDOL inhibition may enhance synaptic functions by modulating Reelin signaling through VLDLR and APOER2. Although we did not assess the effect of *Idol* deletion on tauopathy, previous studies have demonstrated that deletion of *Apoe* and overexpression of *Ldlr* can reduce tau‐associated neurodegeneration.^74^, ^75^ Because IDOL regulates both APOE and LDLR, neuronal IDOL may also influence tau pathology, further underscoring its broad role in multiple AD‐related pathologies. Overall, our results highlight the critical role of neuronal IDOL in AD pathogenesis, providing new therapeutic avenues for AD.

## 5 CONCLUSION

APOE and its receptors play an important role in the pathogenesis of AD. Notably, increasing LDLR levels, either through *Ldlr* overexpression or by deleting its E3 ubiquitin ligase *Idol*, reduced APOE levels and attenuated amyloid pathology.^16^, ^20^ Here, we demonstrate that neuronal, not microglial, IDOL plays a key role in regulating brain APOE and LDLR levels, as well as Aβ accumulation. In addition, neuronal IDOL modulates Reelin receptors, which are critical for synaptic function. Thus, targeting neuronal IDOL may offer multiple therapeutic benefits in AD by simultaneously reducing amyloid burden and gliosis while enhancing synaptic health. Given that IDOL is an E3 ubiquitin ligase and enzymes are highly druggable targets,^85^ its pharmacological inhibition represents a promising strategy for AD treatment.

## AUTHOR CONTRIBUTIONS

Conceptualization: Hande Karahan, Do‐Hun Lee, and Jungsu Kim. Methodology: Hande Karahan, Kelly Hartigan, Md Mamun Al‐Amin, H.R. Sagara Wijeratne, Dominic J. Acri, Luke C. Dabin, Byungwook Kim, Do‐Hun Lee, and Jungsu Kim. Investigation: Hande Karahan, Kelly Hartigan, Md Mamun Al‐Amin, Sutha K. John, Brianne McCord, H.R. Sagara Wijeratne, Dominic J. Acri, Daniel C. Smith, Hannah M. Rondon Cordero, and Do‐Hun Lee. Writing—original draft: Hande Karahan. Writing—review & editing: all authors. Funding acquisition: Jungsu Kim. Supervision: Hande Karahan and Jungsu Kim.

## CONFLICT OF INTEREST STATEMENT

The authors declare no conflicts of interest. Any author disclosures are available in the Supporting Information.

## Supporting information



Supporting Information

Supporting Information

Supporting Information

## Data Availability

Bulk RNA‐seq data have been deposited at GEO: GSE301451 and are publicly available as of the date of publication. Single‐nuclei RNA‐seq data have been deposited at GEO: GSE300690 and are publicly available as of the date of publication. Proteomics data have been deposited at the MassIVE database: MSV000098209 and are publicly available as of the date of publication. All other data generated during this study are included in this published article and its supplementary information files.
