# Supplementary material for: Deletion of neuronal Idol ameliorates Alzheimer's disease–related pathologies via APOE receptors
Source: Alzheimers Dement. 2025 Dec 12;21(12):e70949. doi: 10.1002/alz.70949 (PMC12699379; doi:10.1002/alz.70949)
Supplement: Supplementary file 3 — Supporting Information [file ALZ-21-e70949-s003.pdf]

# Deletion of neuronal *Idol* ameliorates Alzheimer's disease-related pathologies via APOE receptors

Hande Karahan,[1,2] Kelly Hartigan,[1,3] Md Mamun Al-Amin,[1,2] Sutha K. John,[1,2] Brianne McCord,[1,2] H.R. Sagara Wijeratne,[1,4] Dominic J. Acri,[1,3] Daniel C. Smith,[1,3] Luke C. Dabin,[1,2] Hannah M. Rondon Cordero,[1,2] Byungwook Kim,[1,2] Do-Hun Lee,[1,2] Jungsu Kim[1,2,3,*]

## SUPPORTING INFORMATION

**Supplementary Figure S1. *Idol* mRNA is downregulated in the brains of *Idol* conditional knock-out mice**

**Supplementary Figure S2. Soluble Aβ levels were decreased in the cortices of neuronal *Idol* knock-out mice**

**Supplementary Figure S3. Aβ levels in the hippocampus of neuronal *Idol* knock-out mice**

**Supplementary Figure S4. Integrative analysis of transcriptomic and proteomic changes in neuronal *Idol* knock-out mice**

**Supplementary Figure S5. snRNA-seq identifies transcriptional changes and pathway signatures in neuronal populations in neuronal *Idol* knock-out mice**

**Supplementary Figure S6. snRNA-seq identifies transcriptional changes and pathway signatures in other cell populations in neuronal *Idol* knock-out mice**

**Supplementary Figure S7. snRNA-seq identifies a distinct inhibitory neuron population in neuronal *Idol* knock-out mice**

**Supplementary Figure S8. The expression of APOE receptors in neuronal clusters**

## Figure S1.

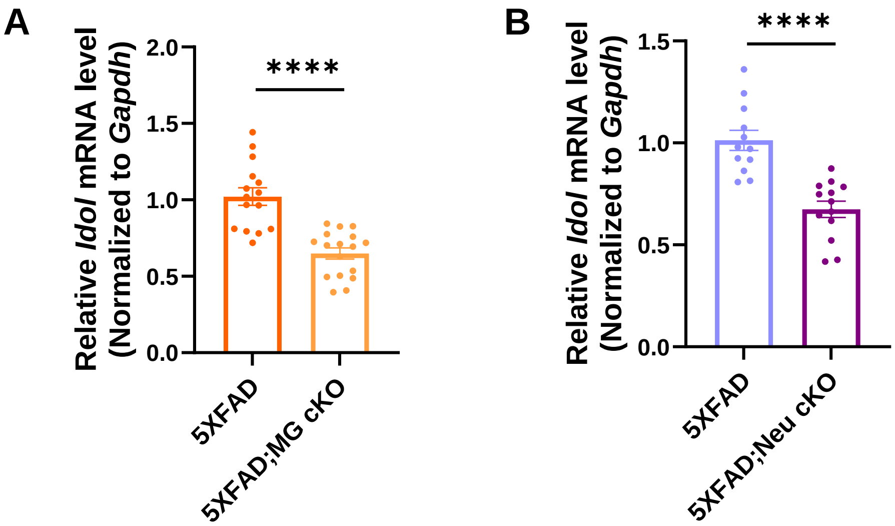


**Supplementary Figure S1. *Idol* mRNA is downregulated in the brains of *Idol* conditional knock-out mice.** *Idol-floxed* mice were bred with *Cx3cr1-CreERT2* or *Camk2a Cre* mice and then with the 5XFAD mouse model to generate microglial (5XFAD;MG cKO) and neuronal (5XFAD;Neu cKO) *Idol* knock-out mice. Microglial *Idol* deletion was induced with tamoxifen injection (75 mg/kg, i.p., 5 days) at 1.5 months of age. *Idol* mRNA levels were measured in the cortices of 4-month-old mice by qPCR. **A)** *Idol* expression was significantly lower in 5XFAD;MG cKO mice compared to littermate 5XFAD controls (N=15-16/genotype). **B)** *Idol* expression was significantly lower in 5XFAD;Neu cKO mice compared to 5XFAD controls (N=11-12/genotype). The data represent mean ± SEM. Unpaired two-tailed t-test; ****p<0.0001.

**Figure S2.**

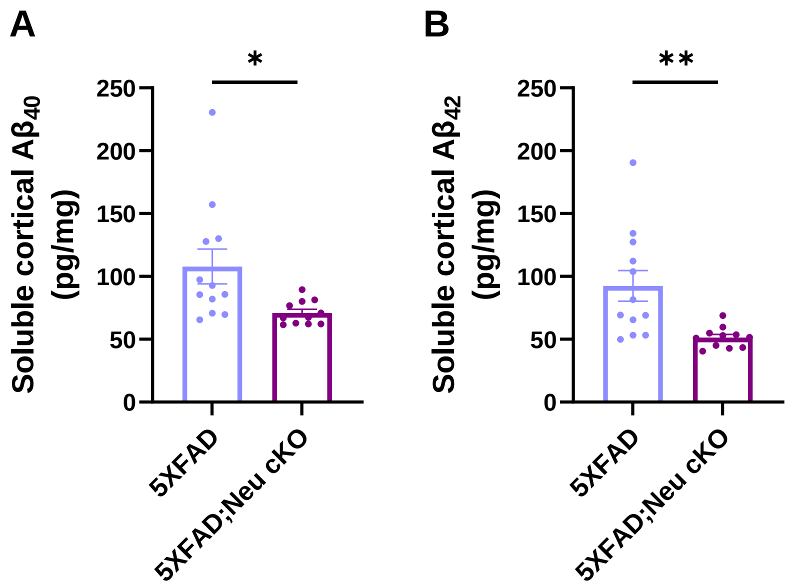


**Supplementary Figure S2. Soluble Aβ levels were decreased in the cortices of neuronal *Idol* knock-out mice.** Soluble Aβ40 and Aβ42 levels were measured in the RIPA fraction of the cortices. **A)** Soluble Aβ40 and **B)** Aβ42 levels were significantly decreased in the cortices of 5XFAD;Neu cKO mice compared to 5XFAD mice (N=11-12/genotype). The data represent mean ± SEM. Unpaired two-tailed t-test; *p<0.05, **p<0.01.

**Figure S3.**

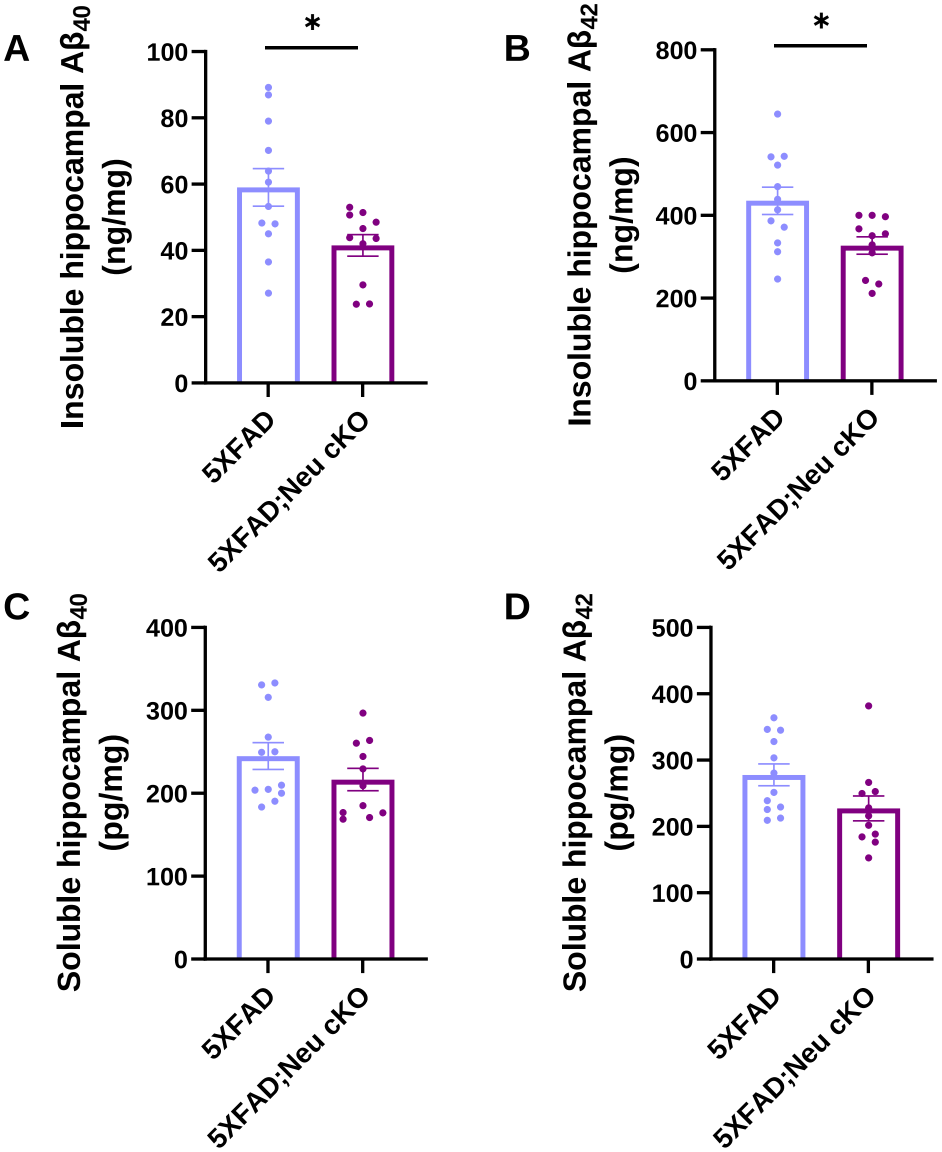


**Supplementary Figure S3. Aβ levels in the hippocampus of neuronal *Idol* knock-out mice. (A-B)** Insoluble Aβ40 and Aβ42 levels were measured in the guanidine fraction of 5XFAD mouse hippocampi using an Aβ electrochemiluminescence assay. **A)** Insoluble Aβ40 and **B)** Aβ42 levels were decreased in the hippocampi of 5XFAD;Neu cKO mice compared to 5XFAD mice (N=11-12/genotype). **(C-D)** Soluble Aβ40 and Aβ42 levels were measured in the RIPA fraction of the hippocampal lysates. **C)** Soluble Aβ40 and **D)** Aβ42 levels were not significantly different between the genotypes (N=11-12/genotype). The data represent mean ± SEM. Unpaired two-tailed t-test; $^{*}p<0.05$.

# Figure S4.

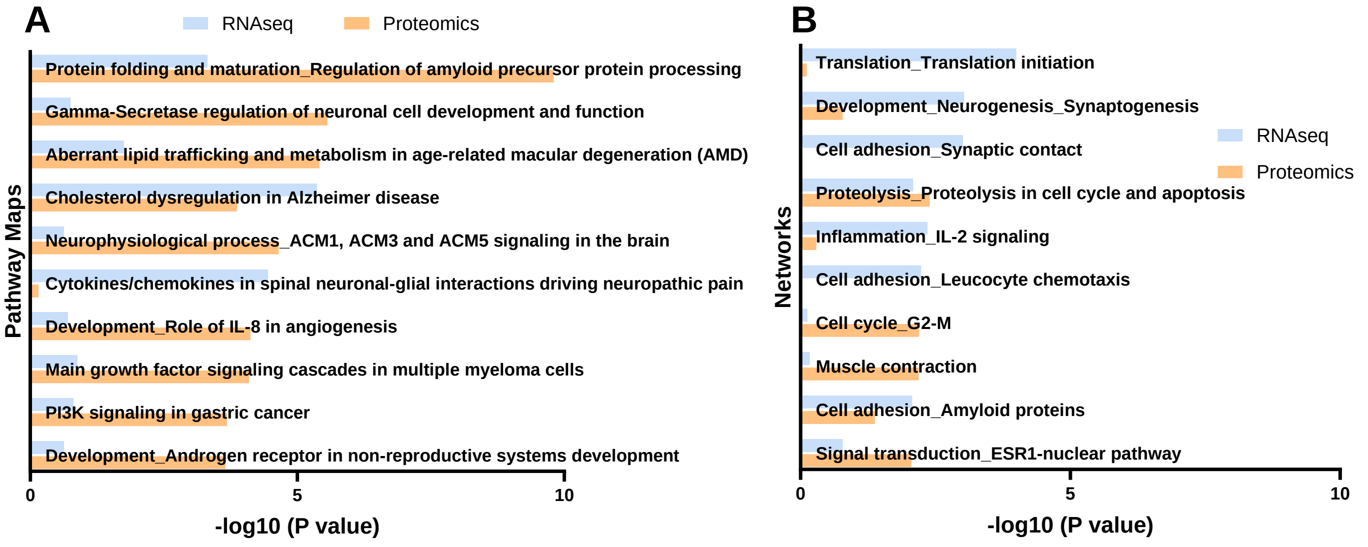


**Supplementary Figure S4. Integrative analysis of transcriptomic and proteomic changes in neuronal *Idol* knock-out mice.** Bulk RNA-sequencing and mass spectrometry were performed on the cortices of 4-month-old 5XFAD;Neu cKO and 5XFAD mice. Differentially expressed genes (DEGs) were identified in bulk RNAseq, and differentially expressed proteins (DEPs) were identified in proteomics. Integrative analyses were performed using DEGs and DEPs with MetaCore™. **A)** Pathway and **B)** network analyses identified enrichment of different pathways driven by transcriptomic or proteomic changes.

# Figure S5.

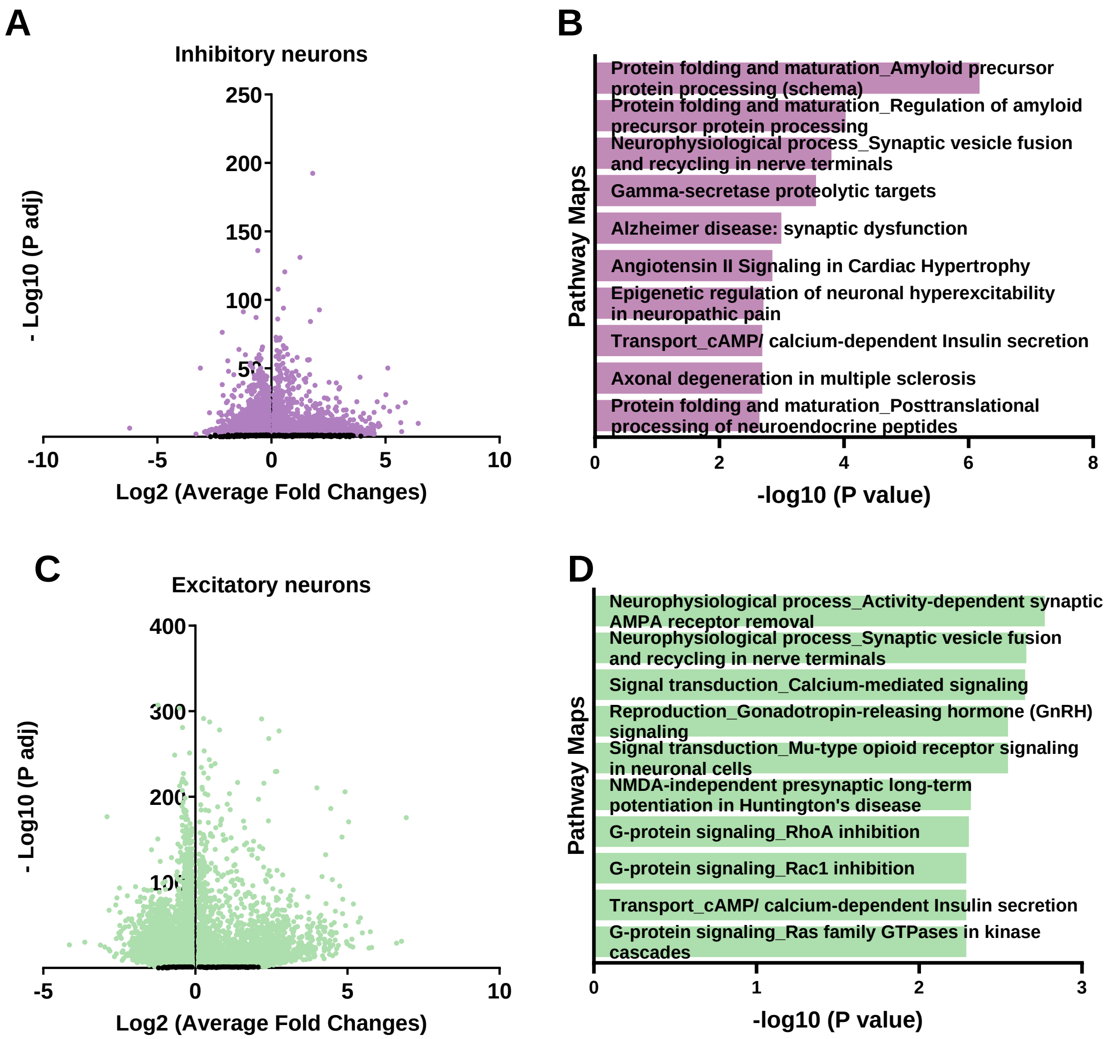


**Supplementary Figure S5. snRNA-seq identifies transcriptional changes and pathway signatures in neuronal populations in neuronal *Idol* knock-out mice. A)** Differentially expressed genes (DEGs) were identified in inhibitory neurons of 5XFAD;Neu cKO mice compared to 5XFAD mice (p-adj<0.05). **B)** Pathway analysis was performed with DEGs using the MetaCore™ software. **C)** DEGs were identified in excitatory neurons of 5XFAD;Neu cKO mice compared to 5XFAD mice (p-adj<0.05). **D)** Pathway analysis was performed with DEGs using the MetaCore™ software. See also Table S9-S12.

# Figure S6.

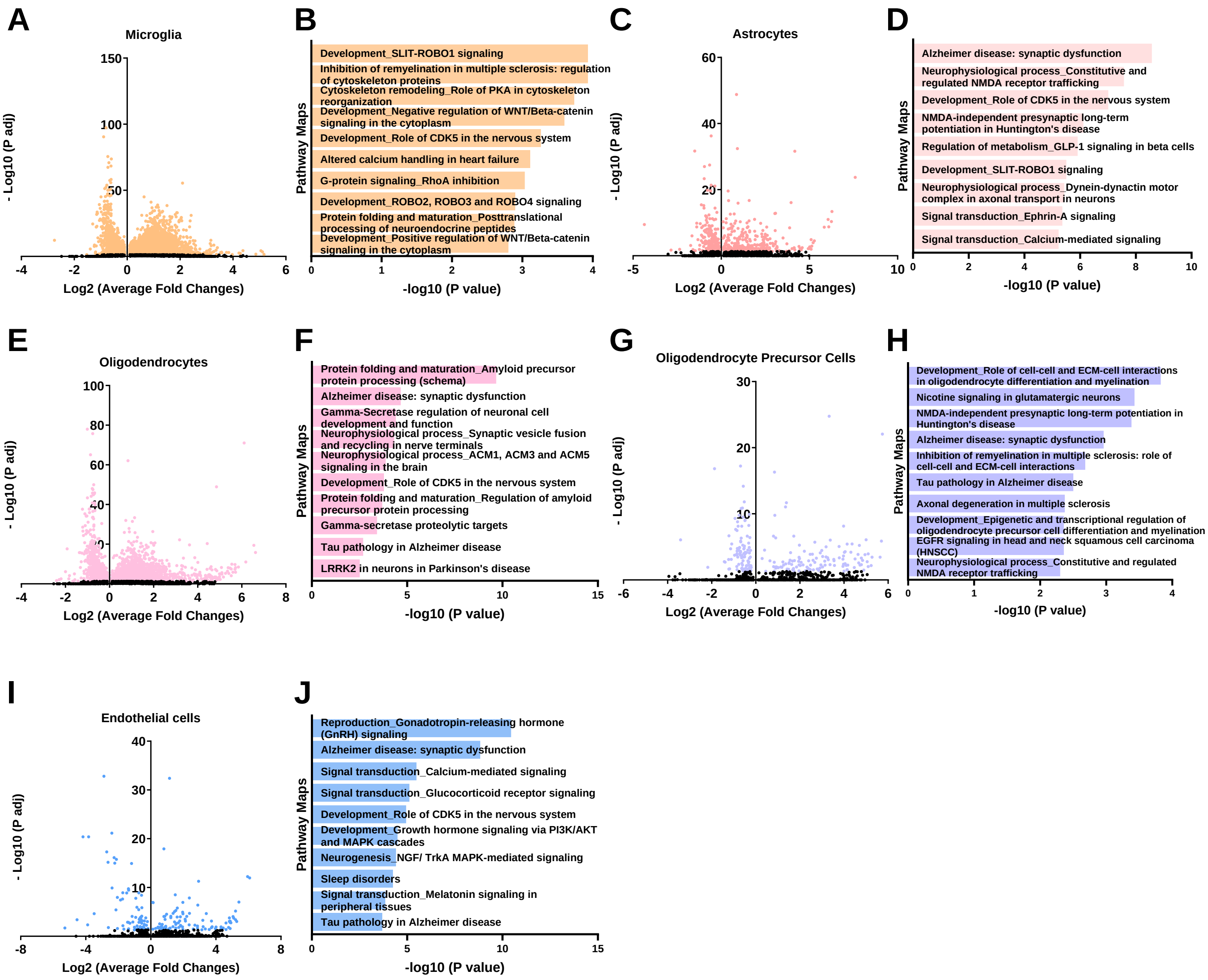


**Supplementary Figure S6. snRNA-seq identifies transcriptional changes and pathway signatures in other cell populations in neuronal *Idol* knock-out mice.** Differentially expressed genes (DEGs) were identified in **A)** microglia, **C)** astrocytes, **E)** oligodendrocytes, **G)** oligodendrocyte precursor cells, and **I)** endothelial cells in 5XFAD;Neu cKO mice compared to 5XFAD mice (p-adj<0.05). Pathway analyses were performed with the DEGs in **B)** microglia, **D)** astrocytes, **F)** oligodendrocytes, **H)** oligodendrocyte precursor cells, and **J)** endothelial cells using the MetaCore™ software. See also Table S13-S22.

**Figure S7.**

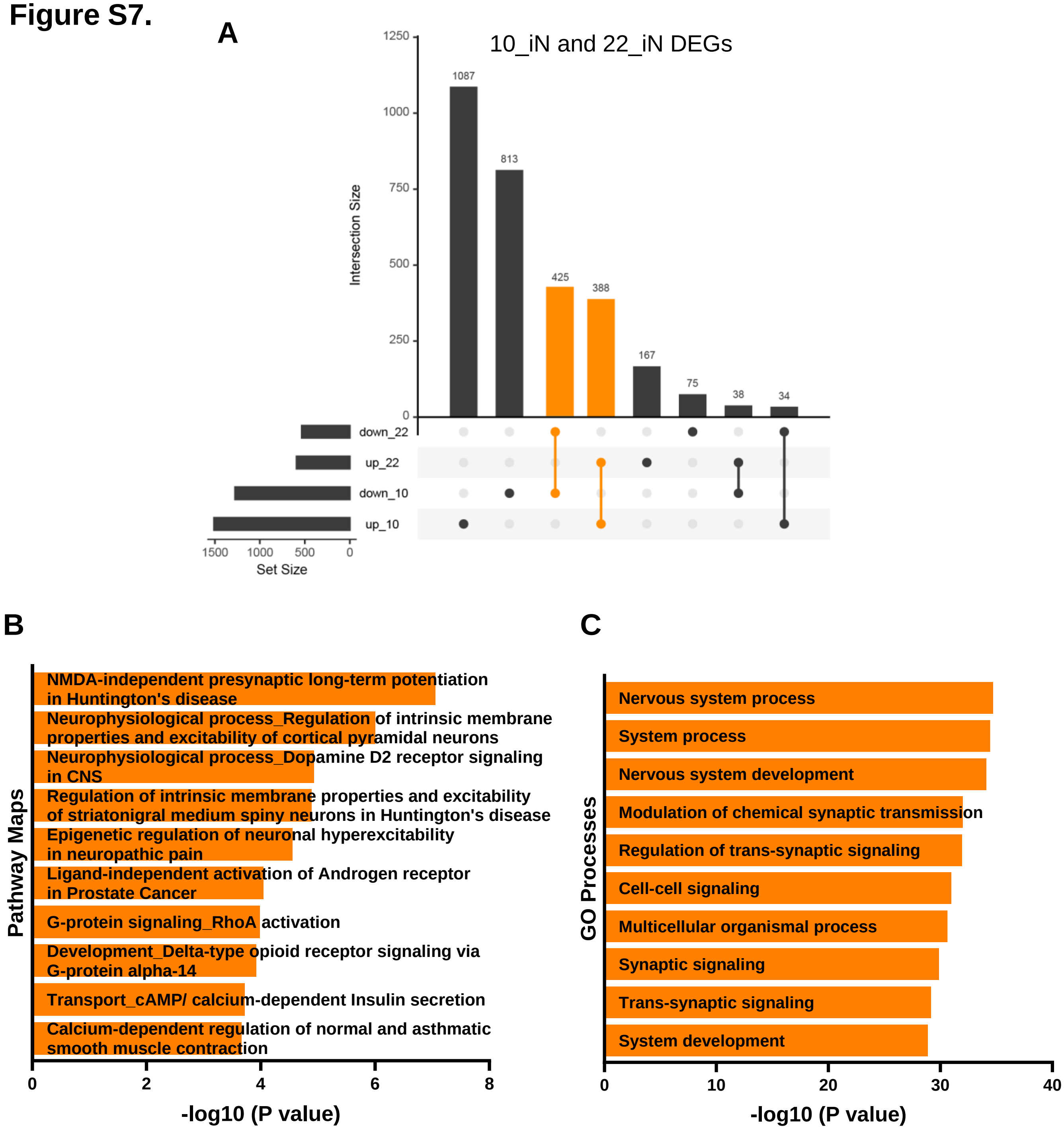


**Supplementary Figure S7. snRNA-seq identifies a distinct inhibitory neuron population in neuronal *Idol* knock-out mice. A)** Differentially expressed genes (DEGs) were identified in *Meis2+* neurons (Clusters 10 and 22) compared to other inhibitory neuron clusters (p-adj<0.05). There were 425 downregulated and 388 upregulated common genes in clusters 10 and 22 compared with the other inhibitory neurons. **B)** Pathway and **C)** GO analyses were performed with the common DEGs in clusters 10 and 22 compared to other inhibitory neuron clusters using the MetaCore™ software. See also Table S24-S26.

**Figure S8.**

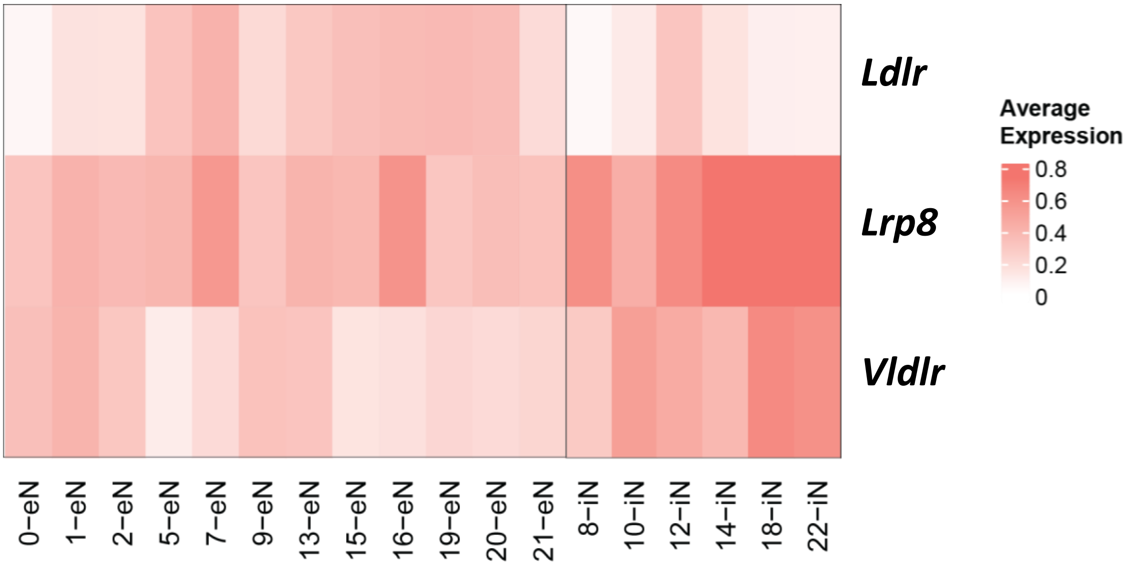


**Supplementary Figure S8. The expression of APOE receptors in neuronal clusters.** The expression of *Ldlr*, *Lrp8* (*Apoer2*), and *Vldlr* is demonstrated in excitatory neurons (eN) (clusters 0-2, 5, 7, 9, 13, 15, 16, 19-21) and inhibitory neurons (iN) (clusters 8, 10, 12, 14, 18, 22) via snRNA-seq analysis. *Lrp8* and *Vldlr* are enriched in inhibitory neurons.
